# Immune-Modulating Dual-Targeted Nanomaterials for Low-Temperature Photothermal–Photodynamic–Chemodynamic Therapy of Osteosarcoma Targeting Tumor and Endothelial Cells

**DOI:** 10.1021/acsnano.5c00223

**Published:** 2025-08-20

**Authors:** Qing Pan, Wei Wu, Doudou Jing, Wei Huang, Yongzhi Cui, Zhicai Zhang, Zengwu Shao, Hongzhi Hu, Wenbo Yang

**Affiliations:** 1 Department of Orthopaedics, Union Hospital, Tongji Medical College, 36630 Huazhong University of Science and Technology, Wuhan 430022, China; 2 Department of Orthopaedics, 74761 Shanxi Medical University Second Affiliated Hospital, Taiyuan, Shanxi 030001, China; 3 Department of Orthopaedics, Shanghai Sixth People’s Hospital Affiliated to Shanghai Jiao Tong University School of Medicine, Shanghai 200080, China

**Keywords:** targeted therapy, biomimetic nanomaterials, photothermal−photodynamic−chemodynamic therapy, immune microenvironment modulation, synergistic therapy

## Abstract

Osteosarcoma is a highly aggressive bone tumor with limited treatment options because of its drug resistance and tumor heterogeneity. In this study, we developed a multifunctional nanomaterial, P-Fe_3_O_4_@Pal@HM, combining porous Fe_3_O_4_ nanoparticles, the DNA intercalator palmatine, and a hybrid membrane coating derived from osteosarcoma cells and tumor-associated endothelial cells. The Fe_3_O_4_ core facilitates a Fenton-like reaction, generating reactive oxygen species (ROS) to enhance DNA damage, whereas palmatine (Pal) inhibits RRM2 (ribonucleotide reductase regulatory subunit M2) expression, blocking DNA repair and inducing apoptosis. The hybrid membrane coating provides precise targeting of both tumor and endothelial cells, thus addressing the challenge of tumor heterogeneity. Under low-temperature photothermal conditions, the Fenton-like reaction is further enhanced, boosting ROS production and increasing cytotoxicity. This nanomaterial also modulates the immune microenvironment by promoting M1 macrophage polarization, thereby amplifying the antitumor immune response. P-Fe_3_O_4_@Pal@HM demonstrated superior therapeutic efficacy to conventional treatments, significantly reducing tumor volume and inducing apoptosis with minimal toxicity to normal tissues. This innovative approach offers a promising strategy to overcome drug resistance, improve tumor targeting, and enhance treatment outcomes in osteosarcoma. The multifunctional design of P-Fe_3_O_4_@Pal@HM highlights its potential as an advanced therapeutic platform in osteosarcoma.

## Graphical abstract

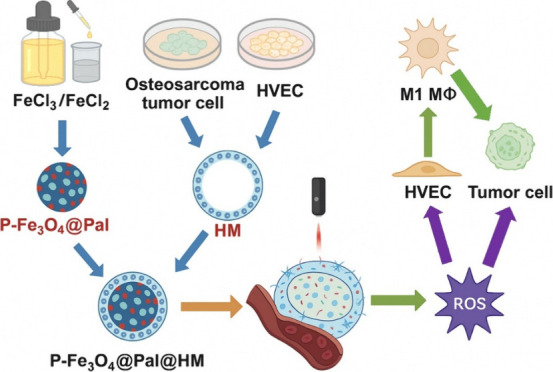

## Introduction

Osteosarcoma is a highly malignant bone tumor that predominantly strikes children and adolescents.^1^ Although the combination of wide surgical resection and multiagent chemotherapy has improved outcomes over the past decades, 5-year survival rates hover around 60–70%, and many patients still experience local recurrence or develop pulmonary metastases.^1,2^ Beyond intrinsic changes within tumor cells—such as gene mutations, upregulated drug-efflux pumps, and alterations in DNA repair pathways—the surrounding microenvironment exerts a decisive influence on therapeutic response.^3^ Histologically, osteosarcoma lesions are a complex ecosystem: alongside malignant osteoblast-like cells, one finds infiltrating immune cells (macrophages, T cells, myeloid-derived suppressor cells) and an extensive vascular network.^3,4^ Importantly, the endothelial cells that line these tumor vessels are not uniform. Single-cell analyses have revealed multiple tumor endothelial subtypes with distinct gene-expression profiles and functional roles. Some subsets promote vessel normalization and drug delivery, whereas others form tight, chemoprotective barriers or secrete cytokines that blunt antitumor immunity.^5,6^ Their marked plasticity and robust capacity for stress-induced repair mean they can survive aggressive chemotherapy and re-establish a protective niche around residual tumor cells. Thus, while most strategies focus on eradicating tumor cells, neglecting these heterogeneous endothelial populations may leave an essential reservoir for relapse. Targeting both malignant osteoblasts and the key stromal architects of chemoresistance—particularly specific endothelial subtypes—could therefore be pivotal to overcoming therapeutic barriers and achieving durable remissions.

Conventional osteosarcoma chemotherapy—most notably cisplatin, doxorubicin, and high-dose methotrexate—targets both rapidly dividing tumor cells and the proliferating endothelial cells that line tumor vasculature.^7^ By inducing DNA cross-links or intercalation, generating ROS, and disrupting mitosis, these agents can damage blood vessels and impair tumor perfusion as well as directly kill malignant osteoblast-like cells.^8,9^ However, this broad cytotoxicity comes at a cost: both tumor and endothelial cells rapidly develop resistance through upregulated drug-efflux pumps, enhanced detoxification enzymes, activation of antiapoptotic pathways, and increased DNA-repair capacity.^10–12^ At the same time, systemic detoxification of these chemotherapeutics places a heavy burden on neural and renal function, leading to cumulative nephrotoxicity and hepatotoxicity that limit dosing intensity and patient tolerance.^13^ Although nanoparticle carriers (e.g., liposomes, polymeric micelles, inorganic scaffolds) have been employed to improve tumor accumulation and reduce off-target toxicity, they often do not fully prevent the emergence of secondary resistance, for the killing mechanism of traditional chemotherapy drugs is relatively limited. In contrast, monomeric small molecules derived from traditional Chinese medicine—particularly isoquinoline alkaloids such as berberine and palmatine—offer multimodal mechanisms (DNA intercalation, ROS generation both spontaneously and upon light activation, and inhibition of DNA repair proteins), a wider therapeutic window with higher lethal dose thresholds in preclinical models, and inherently lower systemic toxicity.^14,15^ These features make TCM monomers attractive candidates for next-generation osteosarcoma therapies, provided their lack of intrinsic tumor and endothelial tropism can be overcome through precision delivery systems.

Nanotechnology offers versatile platforms for overcoming the delivery limitations of small-molecule drugs. Traditional carriers—such as graphene oxide^16^ and polydopamine nanoparticles^17^—excel at drug loading but typically require external triggers (pH shifts, NIR irradiation) to release payloads, leading to high dosage requirements and safety concerns. Iron-containing nanomaterials, especially magnetite (Fe_3_O_4_), stand out by catalyzing Fenton-like reactions in situ, converting endogenous H_2_O_2_ into cytotoxic hydroxyl radicals (chemodynamic therapy) while maintaining stability across physiological pH.^18^ Yet, conventional solid Fe_3_O_4_ nanoparticles possess a low surface area, limited exposure of catalytic iron sites, slow biodegradation, and risk long-term accumulation. Porous Fe_3_O_4_ architectures address these drawbacks through higher specific surface areas that enhance both drug loading and catalytic efficiency, and interconnected pore networks that enable rapid, stimuli-responsive release under acidic or reductive tumor conditions. Despite these advances, porous Fe_3_O_4_ remains “blind” to its cellular targets; without functionalization, it cannot discriminate between malignant osteoblasts, chemoprotective endothelial subsets, or healthy tissue. Therefore, imparting precise targeting motifs—whether via ligand conjugation or biomembrane coating—is essential to direct porous iron oxide nanocarriers selectively to both tumor cells and the endothelial reservoirs of chemoresistance, maximizing therapeutic index while minimizing off-target effects.

Biomembrane coating has emerged as a transformative strategy to confer complex targeting functions onto nanoparticulate cores without laborious chemical conjugations.^19,20^ By cloaking nanoparticles in plasma membranes derived from specific cell types—tumor cells, endothelial cells, and leukocytes—the resulting hybrids inherit the membrane’s native proteins, glycans, and lipid architecture, enabling homotypic adhesion and immune evasion.^21^ Compared to traditional ligand-functionalization approaches (e.g., folic acid, RGD peptides), which require reactive surface groups and risk steric hindrance, biomembrane coatings assemble spontaneously via extrusion or sonication, encasing the nanoparticle core in a biomimetic shell. In this work, we leveraged this platform to construct P-Fe_3_O_4_@Pal@HM: porous Fe_3_O_4_ nanoparticles loaded with palmatine and coated in a hybrid membrane combining osteosarcoma cell and tumor-associated endothelial cell membranes. Palmatine serves as a DNA intercalator and RRM2 inhibitor, disrupting cellular repair pathways in both tumor and endothelial cells. Under NIR irradiation, the porous Fe_3_O_4_ core generates mild photothermal heating and amplifies Fenton-driven ROS production, augmenting palmatine’s cytotoxic effects via combined photodynamic and chemodynamic therapy. The tumor cell membrane component directs nanoparticles to osteosarcoma foci, while the endothelial membrane component facilitates binding to the key vascular subsets that foster chemoresistance. This dual-homing design not only maximizes intratumoral drug delivery but also dismantles the endothelial barrier that protects residual cancer cells, thereby reprogramming the local immune milieu and enhancing overall antitumor efficacy (Scheme 1).

**Scheme 1:**
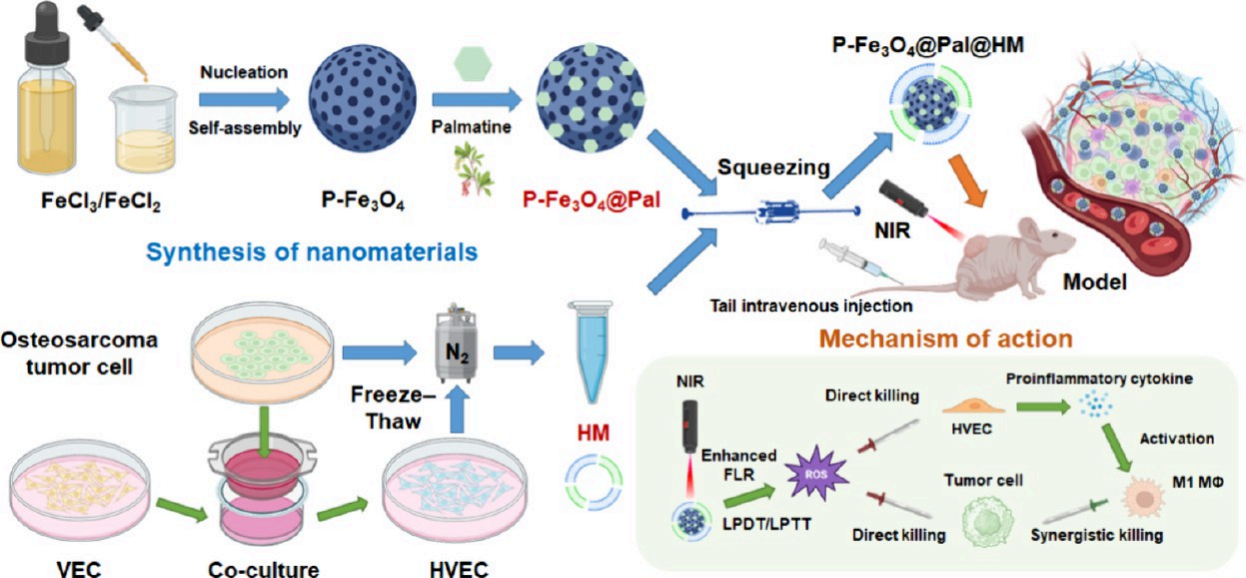
Research Process[Fn sch1-fn1]

a
*Created with BioRender.com.* Pal: palmatine, HM: hybrid membrane, VEC: vascular endothelial cells, HVEC: heterogeneous vascular endothelial cells, NIR: near-infrared radiation, MΦ: macrophage, FLR: Fenton-like reaction, LPDT: low-temperature photodynamic therapy, LPTT: low-temperature photothermal therapy, ROS: reactive oxygen species.

## Results

### Cell Types of Osteosarcoma Lesions and Vascular Endothelium Heterogeneity

We first conducted a study on the pathological characteristics of osteosarcoma cells in order to investigate the causes of osteosarcoma growth, invasion, and frequent treatment resistance, as well as to explore potential therapeutic targets. We initially analyzed the single-cell sequencing results of tissue samples from five primary osteosarcoma lesions. The schematic is shown in Figure 1A. We identified distinct populations of vascular endothelial cells and macrophages within osteosarcoma lesion tissues through dimensionality reduction clustering and annotation (Figure 1B and Figure S1). The vascular endothelial cell population in the osteosarcoma lesion tissues exhibited a PECAM1­(+), CDH5­(+) phenotype, whereas the macrophage population was predominantly of the M2 type, expressing CD163­(+) and MRC1­(+), consistent with the characteristics of the tumor immune microenvironment (Figure 1C). We demonstrated the rich role of vascular endothelial cells in tumor tissues in immune regulation through a circle plot (Figure S2). By introducing normal vascular endothelial tissue as a control group, we used the following criteria for marker selection: genes with an adjusted *p*-value (p_val_adj) < 0.05, avg_log2FC > 0.5 and pct.1 – pct.2 > 0.2, or avg_log2FC < – 0.5 and pct.1 – pct.2 < – 0.2. We discovered that, compared to normal vascular endothelial cells, tumor endothelial cells indeed exhibit heterogeneity, with unique markers of their own (Figure 1D). GO clustering analysis of these markers revealed that the cell membrane of osteosarcoma-associated heterogeneous endothelial cells differs from that of normal endothelial cells, highlighting the potential for specifically targeting osteosarcoma endothelial cells (Figure 1E). In addition, certain intracellular processes, such as nucleotide metabolism, showed significant differences (Figure S3). Subsequently, our CellChat study confirmed the significant regulatory role of osteosarcoma vascular endothelial cells in immune responses (Figure 1F). Heterogeneous vascular endothelial cells can secrete molecules such as MIF and APP, which act on macrophage surface molecules or complexes such as CD74 and CD44, inhibiting macrophage polarization toward the antitumor M1 phenotype and promoting polarization toward the pro-tumor M2 phenotype (Figure 1G). The related mechanism is illustrated in Figure 1H. After coculturing osteosarcoma HVECs with macrophages, we validated this conclusion using PCR, ELISA, and flow cytometry (Figure 1I–P); therefore, in addition to targeting osteosarcoma cells, eliminating osteosarcoma-associated vascular endothelial cells may be a highly necessary strategy for enhancing therapeutic efficacy.

**Figure 1:**
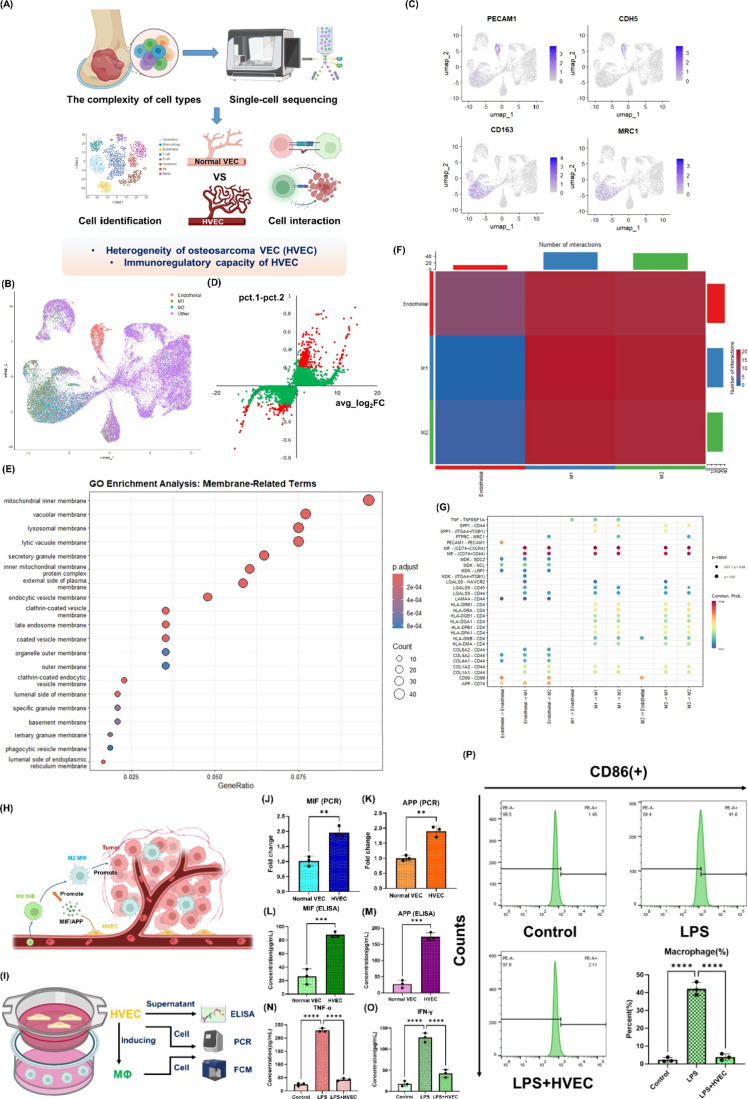
Cell types in osteosarcoma lesions and heterogeneity of the vascular endothelium. (A) Schematic diagram of the single-cell sequencing analysis process of osteosarcoma samples. *Created with BioRender.com.* (B, C) Distribution of vascular endothelial cells and macrophages in osteosarcoma lesions: subpopulations (B) and molecular marker display (C). (D) Gene expression differences between osteosarcoma vascular endothelial cells and normal vascular endothelial cells. pct refers to the proportion of gene expression in the cluster. (E) GO cellular component enrichment analysis of differentially expressed genes, showing the enrichment results of membrane-related pathways. (F, G) Cell interaction results between endothelial cells, M1 macrophages, and M2 macrophages: heatmap (F) and bubble chart (G). The bubble chart displays detailed cell interaction-related molecules. (H) Schematic diagram of the regulatory role of heterogeneous vascular endothelial cells on macrophages. *Created with BioRender.com.* (I) In vitro validation of the regulatory effect of osteosarcoma heterogeneous vascular endothelial cells on macrophages: research schematic. FCM: flow cytometry. *Created with BioRender.com.* (J, K) PCR results of MIF and APP expression levels in macrophages after different inductions. (L, M) ELISA results of MIF and APP secretion levels in macrophages after different inductions. (N, O) PCR results of TNF-α and IFN-γ expression levels in macrophages after different treatments. (P) Flow cytometry results showing the CD86+ ratio (M1 polarization level) in macrophages after different treatments. **p < 0.01; ***p < 0.001; ****p < 0.0001.

### Potential Assessment and Functional Validation of RRM2 as a Therapeutic Target

We conducted sequencing analyses of osteosarcoma tissue samples, osteosarcoma cells, and osteosarcoma-associated heterogeneous endothelial cells to further explore potential therapeutic targets for osteosarcoma and achieve precise and efficient killing of both osteosarcoma cells and osteosarcoma-associated heterogeneous endothelial cells (Figure 2A). By comparing tumor tissues (T group) to adjacent normal tissues (C group), we identified 4465 differentially expressed genes. The volcano plot and heatmap of the key genes are shown in Figure 2B and Figure 2C, respectively. We found that, compared to adjacent normal tissues, osteosarcoma tumor tissues exhibited an overall high expression of DNA metabolism-related genes, such as TOP2A, RRM2, and CLSPN, in most samples, indicating active DNA replication, repair, and other metabolic processes in osteosarcoma. Similarly, genes related to the polarization of macrophages in the anti-inflammatory direction, such as SPP1, MMP13, and SDC1, were significantly upregulated. These gene expression changes may be closely associated with osteosarcoma growth and treatment resistance. In addition, transcriptomic sequencing further confirmed that osteosarcoma cells (Figure 2D) and osteosarcoma-associated heterogeneous endothelial cells (Figure 2E) exhibited significant differences compared to the control groups (osteoblasts/mesenchymal stem cells and normal vascular endothelial cells). GO analysis further confirmed the heterogeneity of osteosarcoma cell membranes compared to controls, as well as the heterogeneity of osteosarcoma-associated heterogeneous endothelial cell membranes compared to controls (Figure 2F,G). The GO (Gene Ontology) biological process also revealed significant differences between osteosarcoma cells and osteosarcoma-associated heterogeneous endothelial cells compared to normal tissue cells. Cellular growth, renewal, and self-repair rely heavily on DNA metabolism. We found that among the DNA metabolism-related genes with elevated expression in osteosarcoma tissue, RRM2 showed a significantly higher expression in both osteosarcoma cells and osteosarcoma-associated heterogeneous endothelial cells (Figure 2D,E,H,I). This indicates that RRM2 plays a crucial role in the heterogeneous proliferation of both osteosarcoma cells and osteosarcoma-associated heterogeneous endothelial cells. The potential interaction targets of RRM2 are shown in Figure S5, most of which are proteins related to cell cycle, DNA metabolism, and cell proliferation pathways. Through Western blot experiments, we found that increased RRM2 expression significantly elevated the expression of cell cycle- and DNA damage repair-related proteins, such as cyclin B1 and CHEK1, in both tumor cells and HVECs (Figure 2J and Figure S4). By analyzing data from TCGA sarcoma cohort, we further confirmed a strong positive correlation between RRM2 and cyclin B1, cyclin E1, and CHEK1 expression, as well as a negative correlation with TP53 expression (Figure S5). In addition, we demonstrated that RRM2 was highly associated with poor prognosis in patients with sarcoma (Figure 2K and Figure S6). These data indicate that the RRM2 gene is highly associated with processes such as cell proliferation, DNA replication, and damage repair. This factor may be a key driver of osteosarcoma cell growth, the gradual development of chemotherapy resistance, and the synchronized rapid growth and chemotherapy insensitivity of osteosarcoma-associated heterogeneous endothelial cells. Therefore, RRM2 could be a highly significant target for simultaneously inhibiting the malignant proliferation of both osteosarcoma cells and osteosarcoma-associated heterogeneous endothelial cells. By directly suppressing tumor growth while inhibiting the proliferation of heterogeneous endothelial cells, RRM2 targeting can enhance osteosarcoma therapy. The related mechanisms are illustrated in Figure 2L. Through flow cytometry, we confirmed that RRM2 overexpression significantly accelerated the cell cycle of both osteosarcoma cells and osteosarcoma-associated heterogeneous endothelial cells, effectively reversing the effects of cisplatin (Figure 2M and Figure S7). These findings further underscore the potential clinical significance of RRM2 as a shared therapeutic target for osteosarcoma cells and associated endothelial cells.

**Figure 2:**
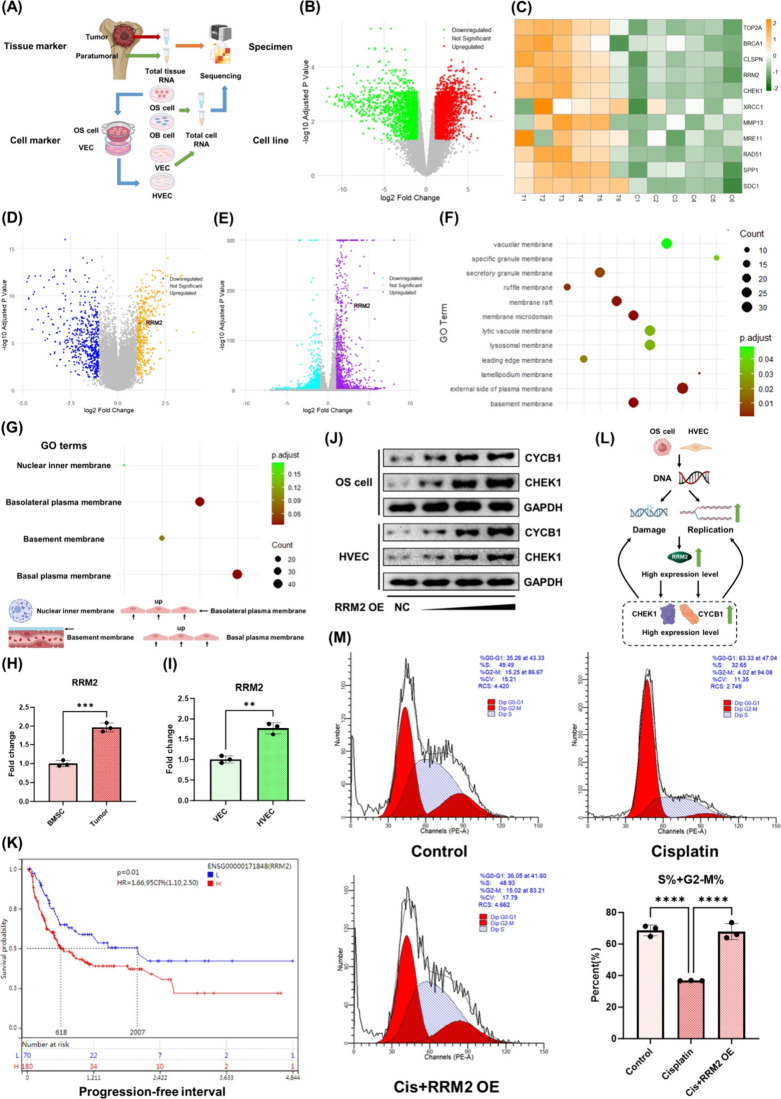
Potential assessment and functional validation of RRM2 as a therapeutic target. (A) Schematic diagram of the study process. OS: osteosarcoma. *Created with BioRender.com.* (B, C) mRNA sequencing results of osteosarcoma tumor tissue (T group) compared to adjacent normal tissue (C group): volcano plot (B) and heatmap of key genes (C). (D) Volcano plot showing differentially expressed genes in osteosarcoma cells compared to osteoblasts/mesenchymal stem cells. RRM2 is upregulated in osteosarcoma cells. (E) Volcano plot showing differentially expressed genes in osteosarcoma-associated heterogeneous vascular endothelial cells compared to normal vascular endothelial cells. RRM2 is upregulated in osteosarcoma-associated endothelial cells. (F) GO cellular component enrichment analysis of differentially expressed genes between osteosarcoma-associated heterogeneous endothelial cells and normal endothelial cells, highlighting membrane-related pathways. (G) GO cellular component enrichment analysis of differentially expressed genes between osteosarcoma cells and osteoblasts/mesenchymal stem cells, focusing on membrane-related pathways. (H, I) PCR validation of increased RRM2 expression in osteosarcoma cells (H) and osteosarcoma-associated heterogeneous vascular endothelial cells (I) compared to normal cells. (J) Western blot validation of increased expression of cell cycle and DNA damage repair-related proteins cyclinB1 and CHEK1 in osteosarcoma cells and HVECs after RRM2 overexpression. OE: overexpression, CYCB1: CyclinB1. (K) TCGA database results show a strong correlation between high RRM2 expression and poor prognosis in patients with sarcoma. Red represents data from patients with high RRM2 expression. The survival cutoff value was set at 5723. (L) Schematic diagram showing the mechanism of action of RRM2. *Created with BioRender.com.* (M) Effect of different treatments on the cell cycle of osteosarcoma cells and the statistical analysis results. ****p < 0.0001.

### Palmatine as an Efficient DNA Intercalative Damage Agent and RRM2 Inhibitor as a Substitute for the Traditional Chemotherapy Drug Cisplatin

RRM2 may be a key molecule responsible for the enhanced DNA metabolism and heterogeneous proliferation of both osteosarcoma cells and osteosarcoma-associated heterogeneous endothelial cells, as confirmed by the aforementioned studies. According to these results, RRM2 was highly associated with active DNA replication and damage repair. Inhibiting DNA replication and damage repair while suppressing RRM2 expression can effectively reduce the self-proliferation of osteosarcoma cells and associated endothelial cells. We discovered that the traditional chemotherapy drug cisplatin, as a DNA intercalator, paradoxically induced increased RRM2 expression at lower concentrations, leading to a self-protective response in cells (Figure S8). Only when cisplatin is applied at high doses does RRM2 expression decrease because of severe DNA damage, leading to cell apoptosis. However, because of cisplatin’s significant toxicity (including its metabolic byproducts), its dosage is often limited, which activates the RRM2-dependent DNA damage repair mechanism. This may explain the limited efficacy of cisplatin and other traditional chemotherapy drugs. Palmatine, a representative isoquinoline alkaloid, contains multiple aromatic rings and a highly conjugated electron system, allowing it to form π–π stacking interactions with DNA base pairs. In addition, isoquinoline alkaloids can efficiently intercalate into DNA through electrostatic interactions and hydrogen bonding, positioning palmatine as a promising alternative to traditional DNA intercalators such as cisplatin. Through molecular docking simulations, we confirmed that palmatine can stably bind to various DNA structures, including G-quadruplex and double-stranded DNA (Figure 3A–C). This binding interaction may effectively inhibit gene replication and transcription. Through database predictions, we found that the promoter sequence of RRM2 contains multiple G-quadruplex structures and AT-rich sequences (Figure S9). Therefore, as a novel DNA intercalator, palmatine can inhibit cell cycle progression by preventing DNA unwinding and replication while simultaneously suppressing RRM2 expression. This mechanism blocks the cell’s self-repair mechanism triggered by increased RRM2 expression. Transcriptomic sequencing of osteosarcoma cells confirmed our database predictions and molecular docking results. The volcano plot and heatmap of the most significantly differentially expressed genes are shown in Figure 3D and Figure S10, respectively. As expected, palmatine significantly reduced RRM2 expression. KEGG enrichment analysis (Figure 3E), GO biological process and cellular component enrichment analysis (Figure 3F,G), and functional analysis of the most significantly differentially expressed genes (Figure 3H) demonstrated that palmatine specifically targets nuclear DNA, strongly inhibiting the cell cycle and affecting DNA damage repair mechanisms. The Western blot results further confirmed that palmatine reduces RRM2 expression in both osteosarcoma cells and HVECs and decreases the expression of cell cycle and DNA damage repair-related proteins, such as cyclin B1, cyclin E1, and CHEK1 (Figure 3I–L). Flow cytometry revealed that palmatine exerted cytotoxic effects on both osteosarcoma tumor cells and osteosarcoma-associated heterogeneous endothelial cells (Figure 3M,N). In clinical applications, due to the lack of targeting specificity of TCM molecules for osteosarcoma cells and osteosarcoma-associated heterogeneous endothelial cells, the intracellular concentration of the drug may not reach optimal therapeutic levels. The mechanism of action and potential limitations of palmatine are presented in Figure 3O. In conclusion, while palmatine effectively inhibits DNA damage repair, metabolism, and cell cycle progression, thereby compensating for the shortcomings of cisplatin, an important area for improvement lies in designing drug delivery systems that can concentrate palmatine at the target site and enhance its cytotoxicity.

**Figure 3:**
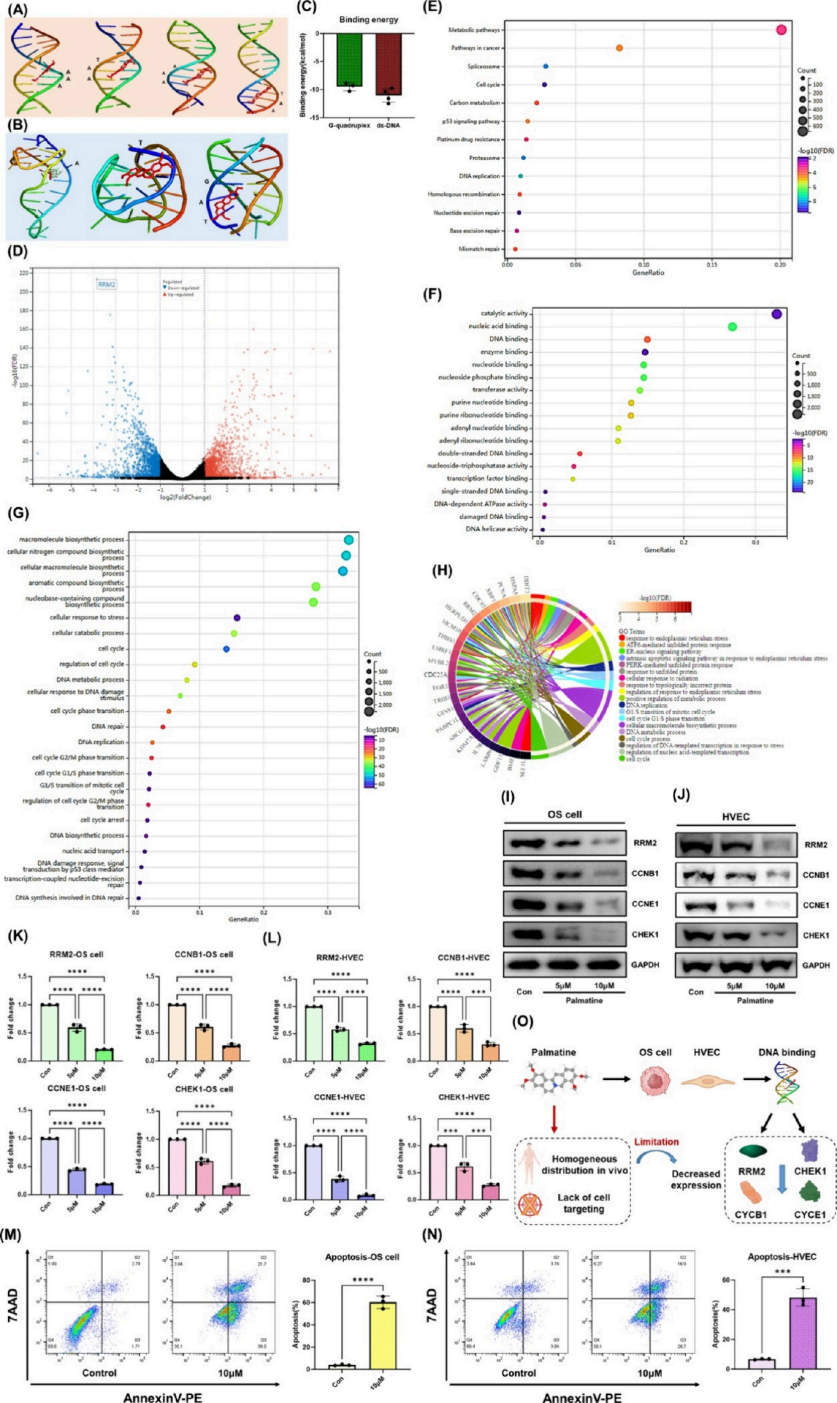
Palmatine as an efficient DNA intercalative damage agent and RRM2 inhibitor as a substitute for the traditional chemotherapy drug cisplatin. (A–C) Molecular docking results of palmatine with AT-rich double-stranded DNA (A) and DNA quadruplex (B), and their calculated binding energies (C). (D) Volcano plot of gene expression changes in osteosarcoma cells after palmatine treatment. (E) KEGG enrichment analysis of differentially expressed genes: bubble plot. (F) GO molecular function enrichment analysis of differentially expressed genes: bubble plot. (G) GO biological process enrichment analysis of differentially expressed genes: bubble plot. (H) GO analysis of key significantly differentially expressed genes: chord diagram. The results show that differentially expressed genes have a significant impact on the cell cycle. (I–L) Changes in the expression levels of DNA metabolism-related gene (RRM2), DNA damage repair gene (CHEK1), and cell cycle-related genes (CCNB1, CCNE1) in osteosarcoma cells (I) and HVECs (L) after treatment with different concentrations of palmatine, with statistical analysis (K, L). (M, N) Apoptosis levels and statistical analysis in osteosarcoma cells (I) and HVECs (L) after treatment with different concentrations of palmatine. (O) Schematic diagram of palmatine’s mechanism of action and potential limitations. *Created with BioRender.com.* ***p < 0.001; ****p < 0.0001.

### Characterization of P-Fe_3_O_4_@Pal@HM Nanomaterials

We designed P-Fe_3_O_4_@Pal@HM nanomaterials to enhance palmatine-induced DNA damage and cell killing effects by utilizing iron-containing nanoparticles to generate ROS via catalysis of Fenton/Fenton-like reactions while also achieving efficient, precise, and sufficient delivery of palmatine. We successfully synthesized the core structure of the composite NPs, P-Fe_3_O_4_. The structure of the nanomaterial and the presence of Fe and O were confirmed through HAADF and elemental analysis (Figure 4A), validating the nanomaterial composition. The loading capacity of Pal by P-Fe_3_O_4_ was subsequently determined to be 35.3%. After loading palmatine, we coated the P-Fe_3_O_4_@Pal nanomaterials with HM. To verify the success of the coating, we performed a low-voltage TEM analysis (Figure 4B). After mixing P-Fe_3_O_4_@Pal with HM using the extruder, a distinct membrane-core structure of P-Fe_3_O_4_@Pal@HM was clearly observed, indicating successful coating. After conducting particle size analysis, we observed an increase in the average particle size of the NPs coated with HM, which further demonstrated the successful synthesis (Figure 4C). The solution stability of the membrane-coated NPs was confirmed through zeta potential analysis, which revealed acceptable results. In addition, the zeta potentials of the membrane-coated NPs were similar to those of the membrane vesicles (Figure 4D). The FTIR and UV–vis results of P-Fe_3_O_4_, Pal, and P-Fe_3_O_4_@Pal (Figure 4E,F) confirmed the successful loading of Pal onto P-Fe_3_O_4_. To further validate the membrane composition, we conducted Western blot experiments to confirm the hybridization of the membranes. In vitro, we obtained the cell membranes of osteosarcoma cells with high expression of CD9-Flag and osteosarcoma-associated HVECs with high expression of PECAM-His, as shown in Figure 4G. The expression of the Flag and His labels was verified after the nanomaterials were coated with a single or hybrid cell membrane (Figure 4H). We observed that the membrane protein components of the P-Fe_3_O_4_@Pal@HM contained CD9-Flag from osteosarcoma cells and PECAM-His from osteosarcoma-associated HVECs, indicating the presence of HM derived from two distinct cell types (Figure 4H). The hybridization success was also confirmed by fluorescent labeling. The experimental process is shown in Figure 4I. Furthermore, we observed that the hybrid membrane-coated NPs exhibited fluorescence of the two colors simultaneously (Figure 4J). The nanomaterial released palmatine in response to the acidic microenvironment of the tumor and the presence of ROS produced by the Fenton-like reaction and NIR. The drug release rates in different environments showed that drug release increased significantly at pH 5.0 and pH 7.4 + H_2_O_2_ + NIR (Figure 4K), with pH 5.0 + H_2_O_2_ + NIR exhibiting the best drug release capability. The targeted release of palmatine has significant implications for enhancing drug concentrations in tumor cells, circumventing the emergence of tumor heterogeneity associated with low-concentration chemotherapy drugs, and mitigating the toxicity associated with cisplatin.

**Figure 4:**
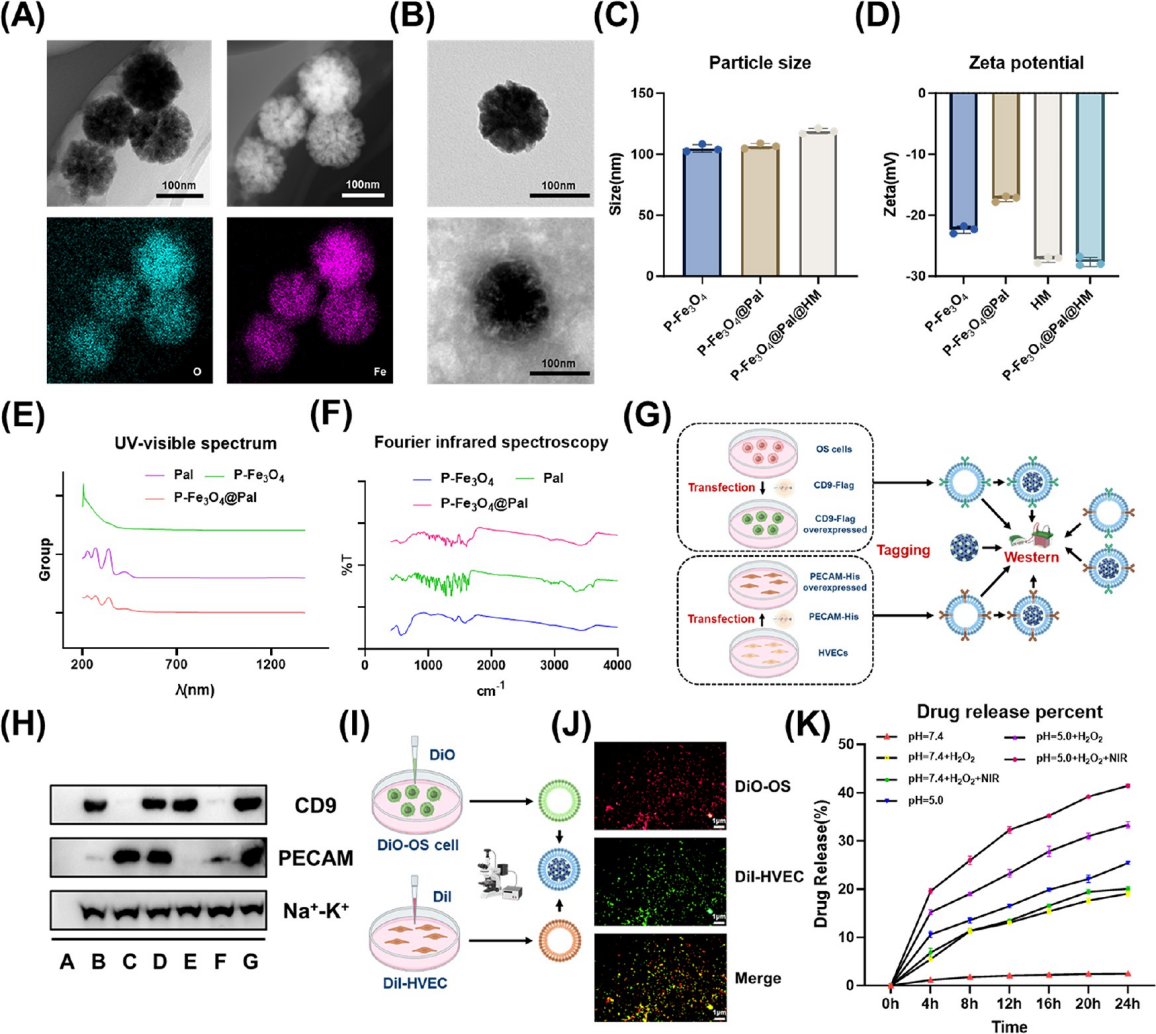
Characterization of P-Fe_3_O_4_@Pal@HM nanomaterials. (A) Transmission electron microscopy (TEM), HAADF, and elemental analysis results of P-Fe_3_O_4_. (B) Low-pressure TEM images of P-Fe_3_O_4_@Pal and P-Fe_3_O_4_@Pal@HM confirm successful membrane coating. (C) Particle size analysis of the nanomaterials. (D) Zeta potential measurement results of the nanomaterials. (E, F) UV–visible spectroscopy (E) and Fourier transform infrared spectroscopy (F) results, confirming successful drug loading. (G, H) Experimental workflow (G) and Western blot results (H) for detecting membrane molecules through membrane marker labeling. *Created with BioRender.com.* (I, J) Experimental workflow (I) and fluorescence microscopy results (J) for observing membrane marker fluorescence in different components. *Created with BioRender.com.* (K) Drug release rate of the nanomaterials under different environmental conditions.

### Biocompatibility of P-Fe_3_O_4_@Pal@HM Nanomaterials

To evaluate the circulatory toxicity of the nanomaterial, we conducted a hemolysis test (Figure 5A,B), as the nanomaterial requires to navigate through the bloodstream to reach the tumor site. Our results indicated that even at a concentration of nanomaterial containing 200 μg/mL of P-Fe_3_O_4_, there was no significant hemolytic activity, suggesting minimal circulatory toxicity. To ensure that the nanomaterial’s effectiveness in targeting osteosarcoma cells did not adversely affect normal bone cells, we assessed its toxicity in normal bone tissue cells. Various concentrations of the nanomaterial were cocultured with osteoblasts, chondrocytes, osteoclasts, and bone marrow mesenchymal stem cells for 24 h, and the cell viability was measured. As shown in Figure 5C–F, the CCK-8 assay revealed no significant toxicity in typical normal bone cells. We also examined the systemic organ toxicity of the nanomaterials in vivo. Histological analysis and HE staining of the major organs (heart, liver, spleen, lung, and kidney) indicated that the tissue morphology of these organs remained unaffected after 14 days of treatment with 20 and 40 mg/kg (calculated based on P-Fe_3_O_4_) of the nanomaterial, and the organs appeared normal after 7 and 14 days of treatment with a dosage of 40 mg/kg, demonstrating low acute and chronic organ toxicity (Figure 5G,H).

**Figure 5:**
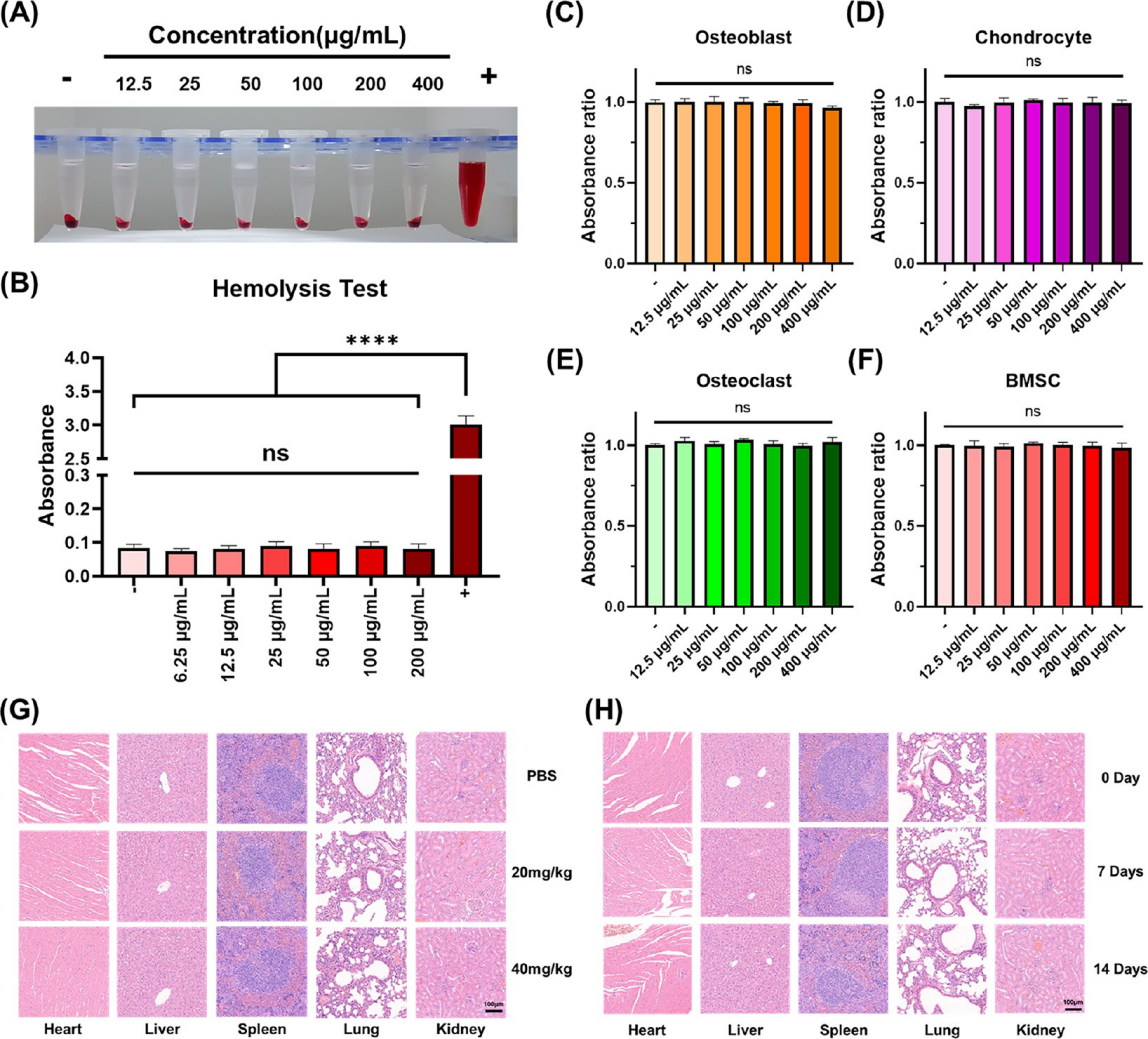
Biocompatibility assessment of the nanomaterials. (A, B) Hemolysis test results and absorbance detection at 541 nm. (C–F) Changes in cell viability after coculture of different concentrations of nanomaterials with osteoblasts (C), chondrocytes (D), osteoclasts (E), and BMSCs (F). (G) HE staining results of major organs after injecting different doses of nanomaterials into animals. (H) HE staining results of major organs at different time points after tail vein injection of 40 mg/kg nanomaterials. ns: nonsignificant. ****p < 0.0001.

### Targeting Ability of P-Fe_3_O_4_@Pal@HM Nanomaterials

Our study shows that P-Fe_3_O_4_@Pal@HM efficiently targets and eradicates HVECs and osteosarcoma tumor cells at the tumor site via the circulatory system. In the xenograft model, P-Fe_3_O_4_@Pal/ICG@HM NPs were found to accumulate in tumors within 8 h while minimizing excessive deposition in vital organs (Figure 6A). Some accumulation was observed in the liver, which is consistent with findings from similar studies and did not affect the functionality of the NPs. To ensure that the fluorescence results are not affected by interference, we used P-Fe_3_O_4_@ICG@HM for most of the in vitro experiments; this model effectively demonstrates the targeting capability of HM. Flow cytometry analysis further validated the cell-targeting capability of the nanomaterials, as shown in Figure 6B. Different cell types were distinguished using a combination of DiD, DiI, and Hoechst 33,342 fluorescent dyes. The labeled cells were mixed and cocultured with NPs for 4 h. Flow cytometry was used to assess the fluorescence levels of tumor cells, HVECs, and normal bone cells. The results indicated significant fluorescence accumulation in osteosarcoma tumor cells and HVECs compared to other normal bone cells (Figure 6C and Figure S11). In addition, we confirmed the uptake of NPs by HVECs and osteosarcoma tumor cells through fluorescence-labeled membrane integration, as shown in Figure 6D. Green fluorescence was used to label the osteosarcoma tumor cell and HVEC cell membranes, whereas the HM of the NPs was labeled with red fluorescence. After 4h of coculture, the cell membranes exhibited both green and red fluorescence, indicating fusion with the NP membrane and subsequent internalization of the nanomaterial (Figure 6E). To further demonstrate the uptake of the nanomaterial by osteosarcoma tumor cells and HVECs, we prepared a fluorescent nanomaterial, P-Fe_3_O_4_@ICG@HM, which labeled only the core of the NPs. After coculture, the red fluorescence of ICG was observed inside the cells, confirming the phagocytosis of nanomaterials by the cells (Figure S12). In comparison, the uncoated nanoparticles P-Fe_3_O_4_@ICG exhibited significantly lower internalization efficiency within the same time frame (Figure S13).

**Figure 6:**
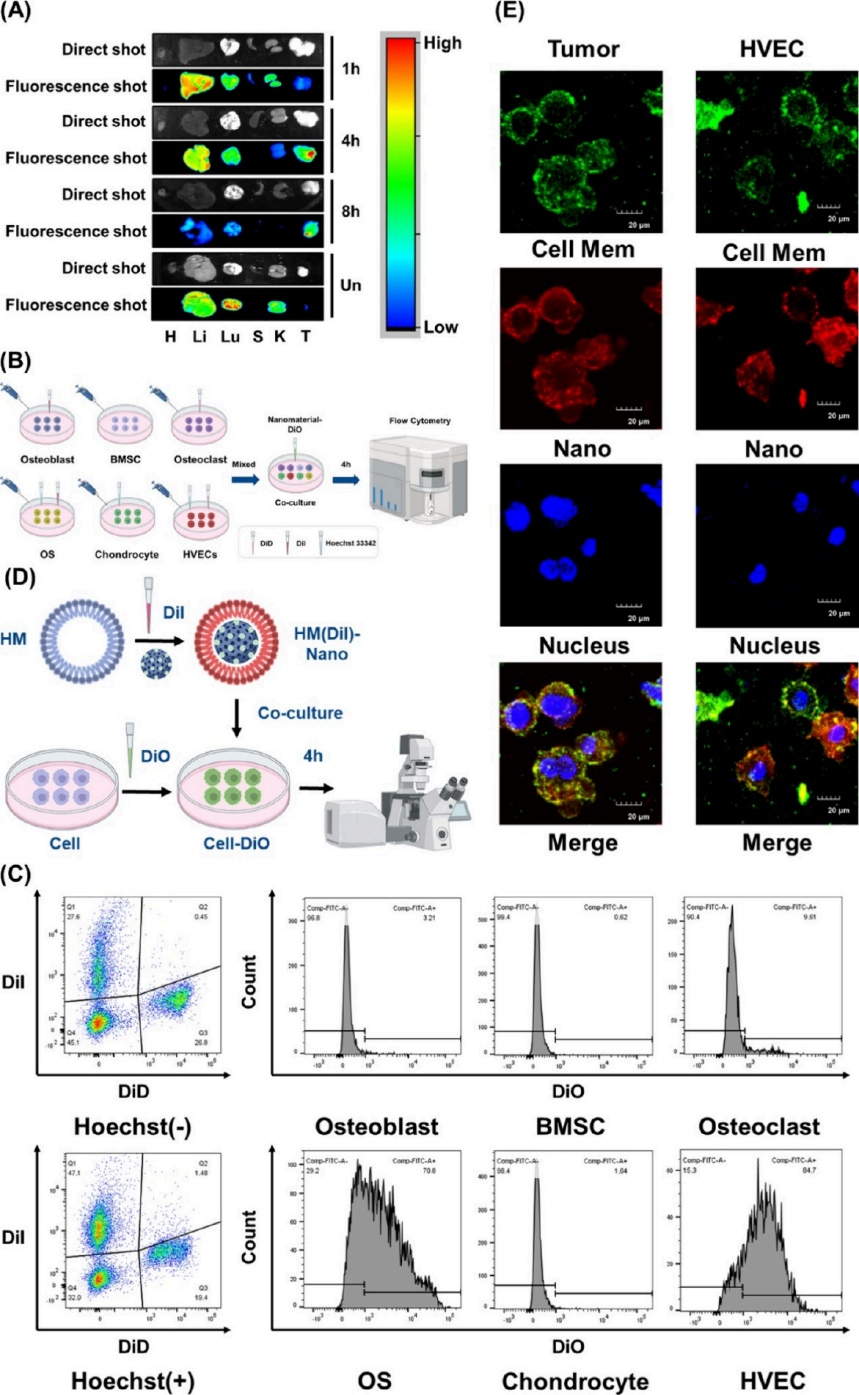
Targeting ability of P-Fe_3_O_4_@Pal@HM nanomaterials (P-Fe_3_O_4_@ICG@HM nanomaterials as the tool of in vitro experiments when necessary). (A) Distribution of P-Fe_3_O_4_@Pal/ICG@HM nanomaterials in the body at different time points after tail vein injection. Un, imaging of uncoated P-Fe_3_O_4_@Pal/ICG nanoparticles 8 h after injection. (B, C) Experimental schematic (B) and flow cytometry results (C) showing the aggregation levels of nanomaterials in different cell types after coculture with cells labeled with different markers. *Created with BioRender.com.* (D, E) Verification of membrane fusion between nanomaterial surfaces and homotypic cell membranes: research schematic (D) and confocal microscopy results (E). *Created with BioRender.com.*.

### Low-Temperature Photothermal Properties of the P-Fe_3_O_4_@Pal@HM Nanomaterials

The photothermal performance of P-Fe_3_O_4_@Pal@HM is primarily attributed to its core structure, namely, P-Fe_3_O_4_. Temperature measurements revealed that P-Fe_3_O_4_, P-Fe_3_O_4_@Pal, and P-Fe_3_O_4_@Pal@HM demonstrated significant photothermal responses (Figure 7A). The photothermal efficiency of the nanoparticles was slightly diminished by the HM coating, likely due to the cell membrane’s resistance to NIR. Nonetheless, the photothermal performance of Fe_3_O_4_@Pal@HM remained adequate. An incremental photothermal response with increasing nanoparticle concentration was observed, as shown in Figure 7B, which led us to select a suitable experimental concentration. For subsequent in vitro studies, a concentration of 100 μg/mL of Fe_3_O_4_ was chosen, as this level is sufficient for low-temperature photothermal therapy. The photothermal stability of Fe_3_O_4_@Pal@HM is confirmed in Figure 7C, with the stability closely linked to the Fe_3_O_4_ core structure. Reliable photothermal stability is crucial for repeated photothermal treatment. In vivo photothermal assessments indicated that the required temperature for low-temperature photothermal therapy was achieved in the tumor region under 1.8 W/cm^2^ NIR irradiation (subsequent studies will maintain the 42.5 °C required for low photothermal temperature therapy) (Figure 7D,E). The thermal stability of the nanoparticles is also critical. Theoretically, NIR irradiation at 42.5 °C should not damage the cell membrane or the structural integrity of the material as this temperature is generally nondamaging to cell membranes and does not degrade other active components of the designed nanoparticles. Our findings confirmed that low-temperature photothermal treatment did not alter the nanoparticles (Figure S14).

**Figure 7:**
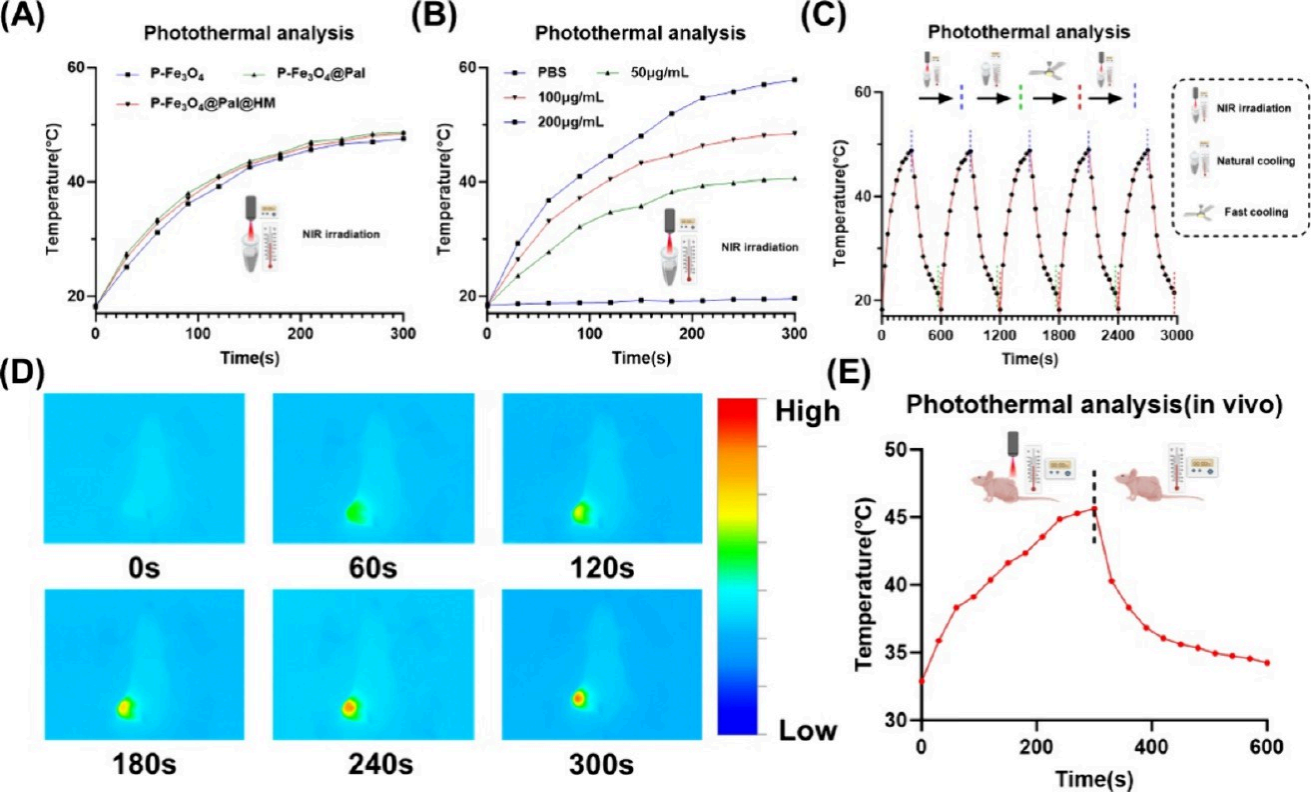
Low-temperature photothermal properties of P-Fe_3_O_4_@Pal@HM nanomaterials. (A) Increase in temperature levels of different nanomaterial components after NIR treatment. (B) Increase in temperature levels of different concentrations of nanomaterials after NIR treatment. (C) Photothermal stability assessment of the nanomaterials. (D, E) In vivo photothermal effects of the nanomaterials (D) and temperature statistics (E).

### P-Fe_3_O_4_@Pal@HM Nanomaterials Combined with Low-Temperature Photothermal Therapy for Direct Killing Heterogeneous Vascular Endothelial and Osteosarcoma Cells

The P-Fe_3_O_4_@Pal@HM complex exerted a significant killing effect on HVECs and osteosarcoma tumor cells, primarily through the generation of ROS via a Fenton-like reaction and the inhibition of DNA damage repair metabolism. We first demonstrated through the TMB assay that the occurrence of the Fenton-like reaction is closely related to the material composition and the acidic tumor microenvironment. This effect was further amplified by low-temperature photothermal/photodynamic effects and effective palmatine delivery (Figure S15). In addition, we also demonstrated the effect of P-Fe_3_O_4_@Pal@HM on DNA damage repair (homologous recombination repair), which is one of the key mechanisms by which the nanoparticles exert cytotoxic effects on target cells (Figure S16). These results provide strong evidence for the efficacy of nanomaterials for selectively targeting heterogeneous blood vessels and drug-resistant tumors. Figure 8A,B shows the results of the CCK-8 assay. The results show that P-Fe_3_O_4_@HM, P-Fe_3_O_4_@Pal@HM, and P-Fe_3_O_4_@Pal@HM + NIR all exhibited cytotoxic effects on these cells, with P-Fe_3_O_4_@Pal@HM + NIR causing the most significant damage. The flow cytometry apoptosis results were consistent with the findings of the CCK-8 assay, indicating that the membrane-coated nanomaterials combined with NIR can effectively kill target cells (Figure 8C). ROS analysis via flow cytometry showed that P-Fe_3_O_4_@Pal@HM and P-Fe_3_O_4_@Pal@HM + NIR significantly elevated intracellular ROS levels, with P-Fe_3_O_4_@Pal@HM + NIR producing the highest levels, corresponding to increased apoptosis (Figure 8D). Increased ROS production is crucial for intensifying DNA damage and mitochondrial impairment. Western blotting and immunofluorescence analyses confirmed that P-Fe_3_O_4_@HM alone could not inhibit RRM2 expression and cell cycle progression. However, P-Fe_3_O_4_@Pal@HM significantly reduced RRM2 expression, with an even more pronounced decrease observed in the P-Fe_3_O_4_@Pal@HM + NIR group. This reduction was associated with severe DNA damage, leading cells to forego repair and enter the apoptosis process. Correspondingly, the Western blot results showed that P-Fe_3_O_4_@Pal@HM + NIR treatment significantly inhibited target cell proliferation and induced apoptosis (Figure 8E–G). We conclude that the increase in ROS levels and inhibition of DNA damage repair resulted in irreparable DNA damage and apoptosis in HVECs and osteosarcoma tumor cells. The P-Fe_3_O_4_@Pal@HM + NIR composite elevated TP53 expression and significantly decreased RRM2 expression, but these changes were partially suppressed by the ROS scavenger NAC in HVECs and osteosarcoma tumor cells (Figure S17). This further demonstrated that the substantial increase in ROS caused severe DNA damage, leading cells to shut down their self-repair mechanisms and enter the apoptotic pathway, which is crucial for eliminating HVECs and osteosarcoma tumor cells. In addition, palmatine helps close the DNA self-repair pathway, thereby significantly enhancing the cytotoxic effect of ROS. The proposed mechanism is illustrated in Figure 8H.

**Figure 8:**
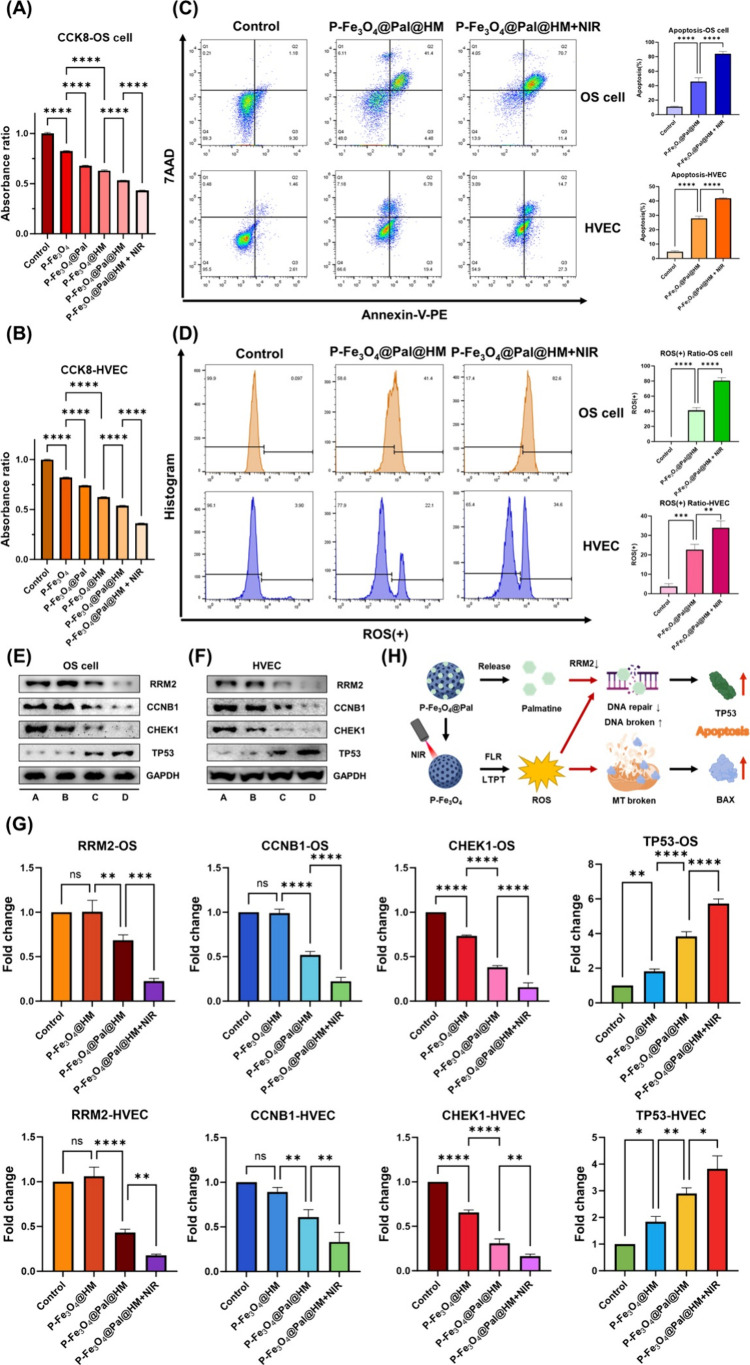
P-Fe_3_O_4_@Pal@HM nanomaterials combined with low-temperature photothermal therapy for direct killing of heterogeneous vascular endothelial cells and osteosarcoma cells. (A, B) Changes in the viability of osteosarcoma cells (A) and HVECs (B) after different treatments. (C) Changes in the apoptosis levels of osteosarcoma cells and HVECs after different treatments and statistical analysis results. (D) Changes in ROS levels of osteosarcoma cells and HVECs after different treatments, and statistical analysis results. (E–G) Western blot results (E, F) and statistical analysis (G) of changes in expression levels of DNA metabolism-related gene (RRM2), DNA damage repair gene (CHEK1), and cell cycle-related genes (CCNB1, CCNE1) in osteosarcoma cells (E) and HVECs (F) after different treatments. (A) Control, (B) P-Fe_3_O_4_@HM, (C) P-Fe_3_O_4_@Pal@HM, and (D) P-Fe_3_O_4_@Pal@HM+NIR. (H) Schematic diagram of the mechanism by which nanomaterials kill target cells after being phagocytosed. *Created with BioRender.com.* MT: mitochondria, ns: nonsignificant; *p < 0.05; **p < 0.01; ***p < 0.001; ****p < 0.0001.

### P-Fe_3_O_4_@Pal@HM Nanomaterials for Regulating the Osteosarcoma Immune Microenvironment: An Enhanced Killing Effect

We previously demonstrated the potential role of HVECs after injury and discussed the necessity of eliminating these cells. Injured HVECs exhibit completely different characteristics to uninjured HVECs. In this section, we confirmed that injured HVECs can induce macrophage polarization toward the M1 phenotype, as illustrated in Figure 9A. Through Transwell coculture experiments, we found that after killing HVECs using P-Fe_3_O_4_@Pal@HM nanomaterials combined with NIR, the expression level of CD86 in cocultured macrophages significantly increased (Figure 9B). PCR, ELISA, and flow cytometry experiments further revealed that injured endothelial cells produced more proinflammatory cytokines, such as TNF-α, IFN-γ, and ROS, which enhanced their antitumor effects (Figure 9C–E). We demonstrated that the aforementioned proinflammatory factors and ROS are the key mechanisms driving macrophage polarization toward the M1 phenotype. Through rescue inhibition experiments, we found that in the coculture system, applying infliximab (MCE, 5 μg/mL), canakinumab (MCE, 5 μg/mL), or NAC (MCE, 3 mM) to the macrophage culture medium weakened the M1 polarization of macrophages (Figure 9F).

**Figure 9:**
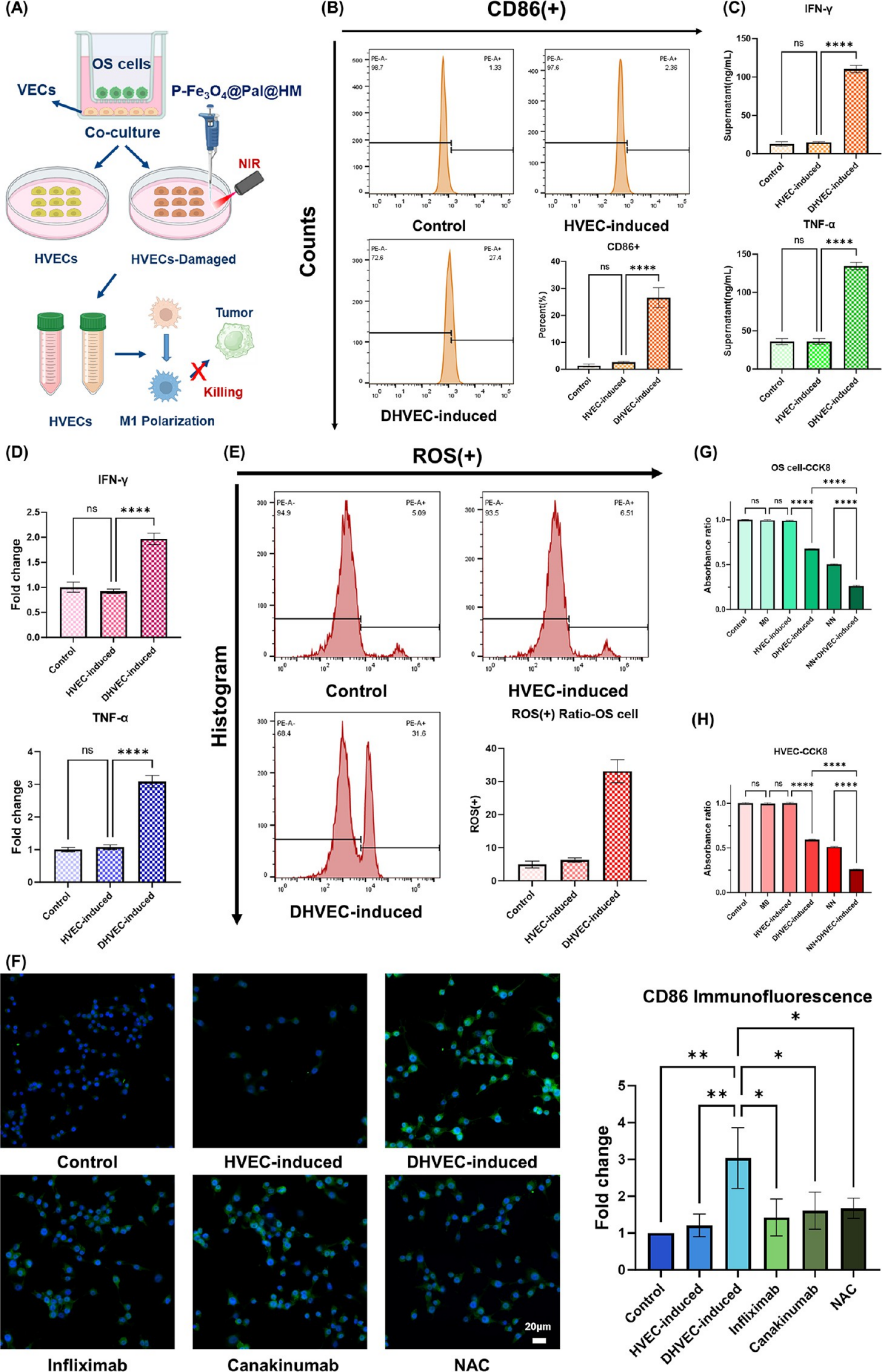

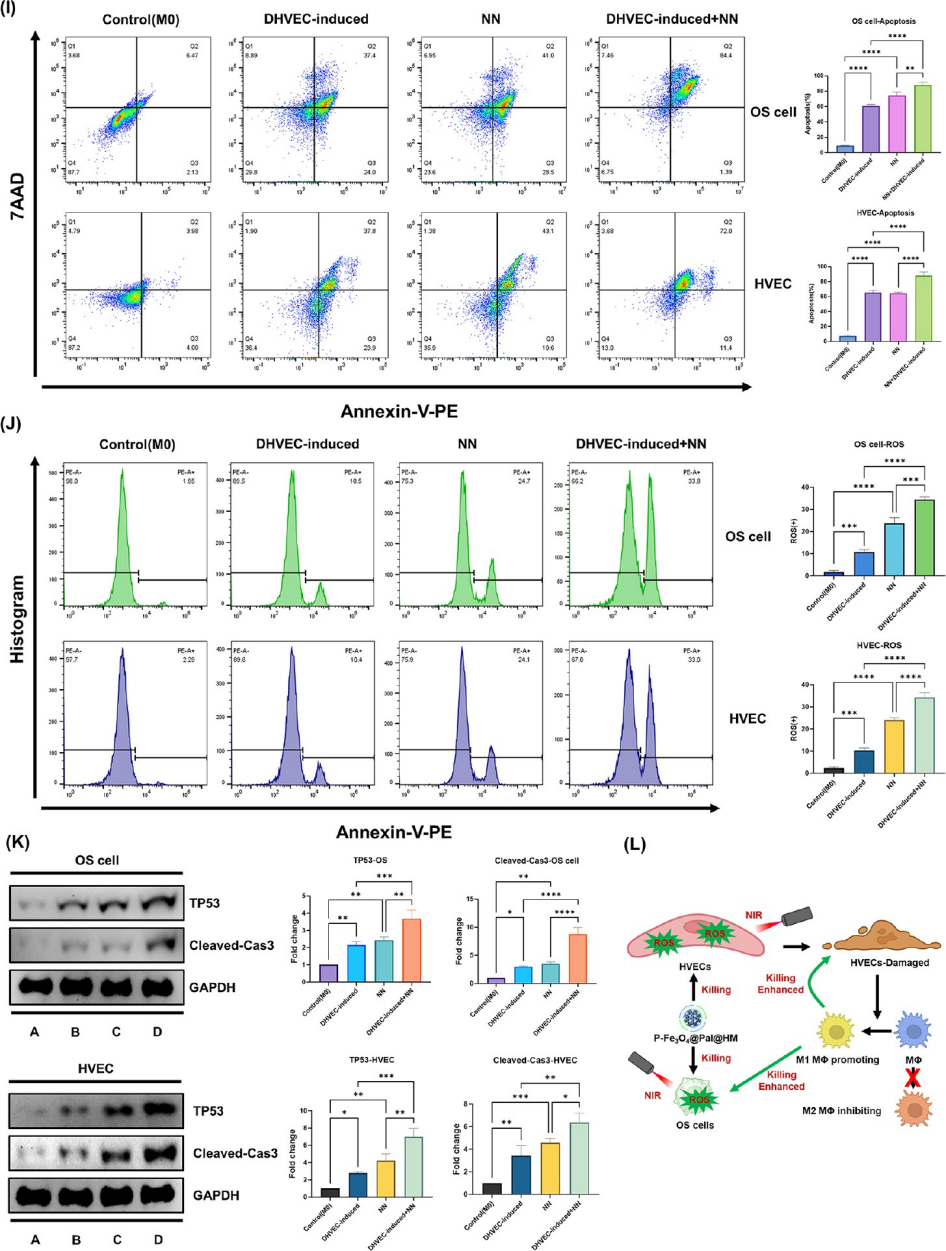
P-Fe_3_O_4_@Pal@HM nanomaterials for the regulation of the osteosarcoma immune microenvironment—achieving an enhanced killing effect. (A) Schematic diagram of the study process. *Created with BioRender.com.* (B) Flow cytometry results and statistical analysis of macrophage M1 polarization levels after coculture with different types of endothelial cells. (C, D) IFN-γ and TNF-α secretion levels in different types of endothelial cells measured by ELISA (C) and PCR (D). (E) Flow cytometry results and statistical analysis of intracellular ROS levels in different types of endothelial cells. (F) Changes in CD86 expression levels on macrophages and fluorescence intensity after different treatments, with statistical analysis. (G, H) Changes in the viability of osteosarcoma cells (G) and HVECs (H) after different treatments. DHVEC: damaged HVEC, NN: nanomaterials combined with NIR. (I) Changes in the apoptosis levels of osteosarcoma cells and HVECs after different treatments, with statistical analysis. (J) Changes in the ROS levels in osteosarcoma cells and HVECs after different treatments, with statistical analysis. (K) Changes in the mitochondrial apoptosis-related proteins TP53 and cleaved-caspase-3 in osteosarcoma cells and HVECs after different treatments, with statistical analysis. (A) Control (cocultured with M0), (B) cocultured with DHVEC-induced MΦ, (C) treated with nanomaterials combined with NIR, (D) cocultured with DHVEC-induced MΦ and treated with nanomaterials combined with NIR. (L) Schematic diagram showing how nanomaterials regulate the immune system to form a positive feedback loop for enhanced therapy. *Created with BioRender.com.* *p < 0.05; **p < 0.01; ***p < 0.001; ****p < 0.0001.

We then investigated how the P-Fe_3_O_4_@Pal@HM nanomaterials regulate the osteosarcoma immune microenvironment, achieving synergistic killing effects on both tumor and heterogeneous endothelial cells. This step creates a positive feedback loop for enhanced tumor therapy. The CCK-8 assay (Figure 9G,H) indicated that the viability of osteosarcoma tumor cells and HVECs remained largely unaffected after coculture with HVEC-stimulated macrophages. However, coculture with supernatant from M1-polarized macrophages induced by damaged HVECs resulted in a significant reduction in cell viability. Under the combined effects of nanomaterials, NIR photothermal therapy, and polarized macrophages induced by injured HVECs, both HVECs and tumor cells exhibited the most significant decline in viability. This apoptotic effect was further amplified when the tumor cells or HVECs were cocultured with nanomaterials paired with NIR. Flow cytometry analysis demonstrated that osteosarcoma tumor cells did not undergo significant apoptosis when cultured with unpolarized macrophages. Apoptosis was noted after coculturing with M1-polarized macrophages induced by damaged HVECs. The extent of apoptosis increased in combination with the expression of M1 macrophages, nanomaterials, and NIR (Figure 9I). The enhanced cytotoxicity toward osteosarcoma tumor cells and HVECs was linked to the secretion of IFN-γ, TNF-α, and other toxic cytokines, along with ROS production by M1-polarized macrophages. The ELISA results revealed that M1-polarized macrophages induced by damaged HVECs secreted high levels of antitumor cytokines such as IL-12, IFN-γ, and TNF-α (Figure S18). ROS level analysis in osteosarcoma tumor cells and HVEC following various treatments showed a significant increase in ROS levels after coculture with M1-polarized macrophages induced by damaged HVECs, and this increase was enhanced by nanomaterials and NIR (Figure 9J). ROS from M1-polarized macrophages also enhanced mitochondrial apoptosis in osteosarcoma tumor cells, as evidenced by increased expression of TP53 and cleaved caspase-3 (Figure 9K). This enhanced apoptosis further suppressed DNA damage repair and reduced RRM2 expression. In summary, the nanomaterials target RRM2 to simultaneously kill HVECs and osteosarcoma tumor cells. The damaged HVECs activate macrophages to polarize toward the antitumor M1 phenotype, resulting in increased ROS production. This leads to more severe DNA damage in both endothelial and tumor cells, further reducing RRM2 expression and diminishing the chances of self-repair in tumor and heterogeneous endothelial cells. This step creates a positive feedback loop for enhanced tumor therapy. The detailed mechanism is illustrated in Figure 9L.

### Evaluation of the In Vivo Therapeutic Effects of P-Fe_3_O_4_@Pal@HM Nanomaterials against Osteosarcoma

We demonstrated in animal models that the nanomaterial P-Fe_3_O_4_@Pal@HM, along with NIR, effectively eliminated tumors by targeting both tumor cells and vascular endothelial cells, as well as promoting M1 macrophage polarization. The therapeutic effect was significantly superior to that of cisplatin or palmatine alone. The experimental workflow is illustrated in Figure 10. The tumor sizes are illustrated in Figure 10B; despite the osteosarcoma’s general insensitive to cisplatin or palmatine alone because of a lack of targeting and synergistic killing ability, significant reductions in tumor volume were observed with P-Fe_3_O_4_@Pal@HM and the addition of NIR. Histological analysis using TUNEL and *K*_i_-67 staining (Figure 10C,D) showed that P-Fe_3_O_4_@Pal@HM combined with NIR resulted in the highest levels of tumor necrosis and apoptosis. When cisplatin or palmatine was used alone for treatment, only limited tumor suppression was achieved, corresponding to the tumor size. RRM2 staining of the tumor tissue (Figure 10E) revealed that cisplatin treatment alone barely reduced RRM2 expression within the tumor, whereas palmatine partially reduced RRM2 expression. However, due to the lack of targeting, its efficiency was still limited. P-Fe_3_O_4_@Pal@HM combined with NIR fully amplified palmatine’s effects, effectively inhibiting RRM2 expression in tumor tissue. Further staining for CD31 (Figure 10F) and CD86 (Figure 10G) showed a reduction in blood vessel density and an increase in M1 macrophages after treatment with P-Fe_3_O_4_@Pal@HM combined with NIR. A significant reduction in blood vessel density occurs only when endothelial cells are precisely targeted and killed. Similarly, although cisplatin and palmatine exerted inhibitory effects on the tumor, they only mildly activated local antitumor immunity. In contrast, combined treatment with nanomaterials and mild photothermal therapy significantly promoted M1 macrophage polarization after endothelial cell killing in vivo, clearly demonstrating the enhanced therapeutic effect. To verify the in vitro mechanism, macrophages, HVECs, and tumor cells were isolated from the xenografts using magnetic bead separation, and RNA was extracted for PCR (Figure 10H). Post-treatment with P-Fe_3_O_4_@Pal@HM and NIR, macrophages exhibited increased NF-κB expression (Figure 10I), alongside heightened levels of TNF-α, indicating a shift toward the M1 phenotype (Figure 10J). The expression of RRM2 in both HVECs and tumor cells was significantly reduced, which is associated with the action of palmatine, further confirming the treatment’s efficacy (Figure 10K–L). Subsequently, through in vivo experiments, we demonstrated that RRM2 is a key therapeutic target of P-Fe_3_O_4_@Pal@HM and NIR (Figure 10M). Notably, when RRM2 was overexpressed in tumor cells and HVECs, tumor growth was significantly accelerated, and the therapeutic effects of P-Fe_3_O_4_@Pal@HM and NIR were suppressed. These findings highlight the effectiveness of P-Fe_3_O_4_@Pal@HM combined with NIR for treating osteosarcoma by directly targeting and killing tumor cells and endothelial cells through mitochondrial apoptosis and inhibition of DNA repair while simultaneously inducing macrophage polarization to achieve synergistic tumor cell destruction.

**Figure 10:**
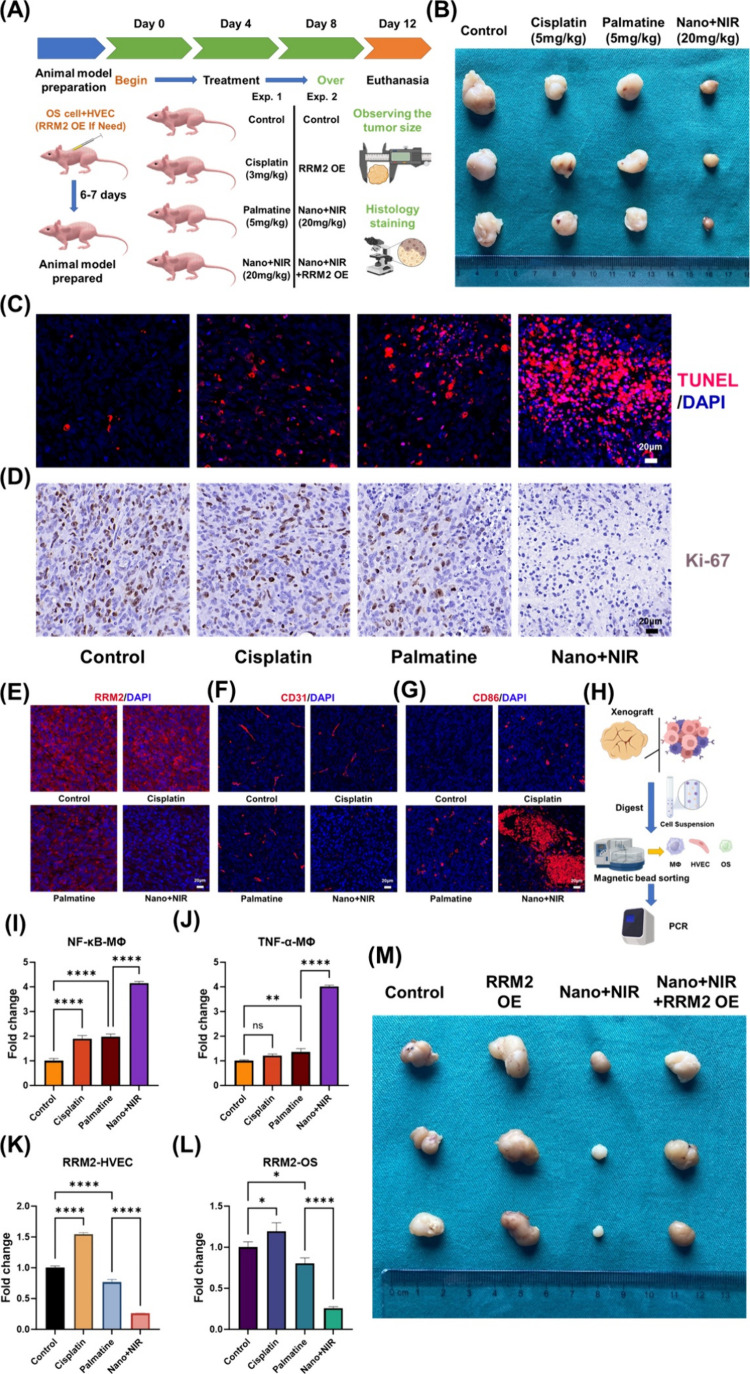
Evaluation of the in vivo therapeutic effects of P-Fe_3_O_4_@Pal@HM nanomaterials combined with NIR for osteosarcoma. (A) Schematic diagram of the study process. *Created with BioRender.com.* (B) Comparison of tumor volumes after different treatments. (C, D) TUNEL staining results (C) and *K*_i_-67 staining results (D) of tumors after different treatments. (E–G) Staining levels of RRM2 (E), CD31 (F), and CD86 (G) in tumor tissues after different treatments. (H) Schematic diagram of the study process for extracting different cells from tumor tissues and performing intracellular RNA PCR detection. *Created with BioRender.com.* (I, J) Expression levels of NF-ΚB (I) and TNF-α (J) in macrophages within tumor tissues. (K, L) Expression levels of RRM2 in HVECs (K) and tumor cells (L). (M) In vivo experimental validation of RRM2 as a key therapeutic target. *p < 0.05; **p < 0.01; ****p < 0.0001.

## Discussion

Osteosarcoma, as a highly malignant solid tumor, not only comprises tumor cells but also includes a variety of stromal cells, such as vascular endothelial cells, tumor-associated fibroblasts, and tumor-associated macrophages.^22^ These stromal cells exhibit significant functional and genetic heterogeneity, which profoundly affects tumor growth, metastasis, and chemotherapy resistance. In our study, single-cell sequencing of tissue from five primary osteosarcoma lesions identified the presence of various stromal cell types. Specifically, the vascular endothelial cells in osteosarcoma were characterized by the expression of PECAM1­(+), CDH5­(+), and VWF­(+), whereas the macrophages were predominantly of the M2 type, expressing CD163­(+) and MRC1­(+). This expression pattern is highly consistent with the immunosuppressive characteristics of the tumor microenvironment.^23^ The heterogeneity of vascular endothelial cells and macrophages plays a crucial role in various functions within the tumor microenvironment. For example, vascular endothelial cells can promote tumor angiogenesis by supplying nutrients and oxygen to tumor cells, thereby supporting their growth.^24^ Tumor-associated macrophages, particularly M2-type macrophages, support tumor immune evasion and promote drug resistance by secreting anti-inflammatory cytokines, which inhibit the activation of antitumor immunity.^25,26^ In this study, we confirmed that osteosarcoma-associated HVECs not only play a structural role in transporting nutrients and supporting tumor growth but also serve a functional role in promoting tumor growth by regulating the tumor immune microenvironment. This functional role is primarily attributed to the heterogeneity of osteosarcoma-associated endothelial cells. The heterogeneity of tumor vascular endothelial cells has been preliminarily explored in some studies.^27^ However, research on the molecular biology characteristics, immune-regulating functions, and potential therapeutic targets of osteosarcoma-associated HVECs is lacking. In this study, by comparing vascular endothelial cells from osteosarcoma to normal endothelial cells, we discovered that osteosarcoma-associated endothelial cells exhibit unique heterogeneity, with significant differences in their membrane markers and intracellular metabolic activities. GO analysis revealed that these heterogeneous endothelial cells show marked differences compared to normal endothelial cells, particularly in enhanced activities related to nucleotide metabolism and DNA repair. These functional differences may be key factors contributing to the insensitivity of osteosarcoma-associated heterogeneous endothelial cells to traditional cisplatin treatment regimens. In addition, through CellChat analysis, we found that HVECs in osteosarcoma secrete molecules such as MIF and APP, which interact with macrophage surface molecules or complexes such as CD74 and CD44. This interaction inhibits macrophage polarization toward the M1 phenotype and promotes the formation of M2-type macrophages. To validate these findings, we conducted in vitro experiments by coculturing osteosarcoma-associated HVECs with macrophages. The results illustrated that osteosarcoma-associated endothelial cells significantly promoted macrophage polarization toward the M2 phenotype while inhibiting the formation of M1-type macrophages. This phenomenon indicates that heterogeneous endothelial cells in osteosarcoma not only promote tumor angiogenesis but also enhance immune evasion and drug resistance by regulating immune cell behavior. These results highlight the necessity of targeting and eliminating heterogeneous endothelial cells, in addition to tumor cells, for effective osteosarcoma treatment. To further explore potential therapeutic targets for osteosarcoma, we conducted a detailed analysis of transcriptome sequencing results for osteosarcoma tissues and their associated HVECs. The analysis revealed that genes related to DNA metabolism, such as RRM2, were significantly overexpressed in osteosarcoma tissues. Notably, after treatment with chemotherapy drugs such as cisplatin, the expression of RRM2 was further elevated. This suggests that RRM2 plays a critical role in DNA replication and repair, thereby providing strong growth and repair capabilities to both tumor cells and vascular endothelial cells within the tumor microenvironment. Prior studies have highlighted the important role of RRM2 in tumor growth and chemotherapy resistance.^28^ In this study, we confirmed that high RRM2 expression is not only closely associated with tumor cell proliferation and survival but also plays a key role in damage repair. In cell experiments, knockdown of RRM2 significantly reduced the DNA damage repair capacity of osteosarcoma cells and their associated vascular endothelial cells, leading to marked cell cycle arrest, which resulted in spontaneous damage and apoptosis due to the rapid proliferation of the cells. These findings suggest that RRM2 is a highly promising therapeutic target. Intervening its function can simultaneously inhibit malignant proliferation and self-repair of both tumor cells and vascular endothelial cells, thereby improving therapeutic outcomes in osteosarcoma.

We are eager to find a replacement for cisplatin, as its high toxicity—both from the drug itself and its metabolites—poses significant challenges. In addition, the self-protection mechanisms induced by this agent in cells have become a major limitation to its application in osteosarcoma treatment.^29^ Based on current research, precision therapies targeting the heterogeneous DNA damage–repair processes within both tumor cells and tumor-associated stromal cells remain a pivotal strategy.^30^ Finding a replacement drug that can effectively bind to DNA and inhibit DNA transcription, replication, and related metabolic activities while also suppressing the expression of key DNA damage repair proteins would be an ideal solution. In osteosarcoma, because of the critical role of RRM2 in both tumor cells and tumor-associated HVECs, it is particularly important to simultaneously damage DNA and inhibit RRM2 expression for effective treatment.^31^ In this study, we explored the potential of palmatine, an isoquinoline alkaloid, as a cisplatin substitute. Through molecular docking and in vitro experiments, we found that palmatine intercalates into DNA and inhibits RRM2 expression, effectively blocking the DNA repair pathway and thereby enhancing the effects of chemotherapy. Our experimental results demonstrated that palmatine not only prevents DNA replication and repair, limiting cell proliferation, but also significantly reduces RRM2 expression, inhibiting osteosarcoma cell and vascular endothelial cell growth. However, due to the low intracellular delivery efficiency of palmatine and its limited inherent cytotoxicity, it primarily exhibits inhibitory effects on cell proliferation rather than direct cell killing. Without specific targeted delivery and enhanced cytotoxicity, the killing effect is limited. This suggests the need for further optimization of palmatine delivery systems in clinical applications to achieve more effective antitumor outcomes.

The traditional chemotherapy drug cisplatin causes DNA damage not only through its ability to form cross-links with DNA but also by generating reactive oxygen species (ROS), which is another key mechanism of its action.^32^ However, because of the limitations in targeting and the toxicity of the drug, the concentration of cisplatin in the tumor microenvironment is often insufficient. As a result, ROS generation is frequently inadequate, which in turn activates the cell’s self-repair mechanisms rather than effectively inducing cell death.^32^ Theoretically, excessive ROS production can cause severe cellular damage and induce apoptosis. In recent years, Fenton–Fenton-like reactions, which are powerful tools for ROS generation, have been preliminarily explored in tumor therapy research.^33^ Catalyzing Fenton-like reactions with ferric ion-containing nanoparticles to generate excessive ROS has been shown to effectively induce DNA damage, making it a prominent example of chemodynamic therapy.^34^ Furthermore, Fenton-like reactions can be enhanced with increasing temperature, providing the necessary conditions for low-temperature photothermal therapy to synergistically boost chemodynamic therapy.^35^ In this study, to enhance the delivery efficiency of palmatine and leverage the excessive ROS generated by low-temperature photothermally enhanced Fenton-like reactions to further increase the killing effect on tumor cells and vascular endothelial cells, we designed porous P-Fe_3_O_4_ nanoparticles for palmatine loading. The mesoporous structure of the Fe_3_O_4_ nanoparticles provides a very high specific surface area and tunable pore size, enabling efficient loading and sustained release of palmatine to boost local drug concentration in the tumor microenvironment.^36^ Meanwhile, the component of nanoparticles, porous architecture, and exposed crystal facets synergistically enhance Fenton-like activity by accelerating H_2_O_2_ diffusion and electron transfer, generating abundant ·OH radicals for increased cytotoxicity against tumor cells and HVECs.^37^ The experimental results showed that the porous structure of P-Fe_3_O_4_ nanoparticles allows for high drug loading efficiency. However, P-Fe_3_O_4_ nanoparticles alone lack specificity for tumor cells and osteosarcoma-associated HVECs (HVECs) and thus fail to achieve targeted killing. In this study, we chose hybrid cell membranes as a means of targeting the target cell. In addition to homotypic targeting capabilities, hybrid cell membranes also express ″do not eat me″ signals such as CD47, making the nanoparticles more favorable for accumulation in target cells and minimizing toxicity.^38^ In this study, by characterizing the nanomaterials, we confirmed that P-Fe_3_O_4_@Pal@HM nanomaterials can be synthesized easily and stably. The P-Fe_3_O_4_@Pal@HM nanomaterials effectively released palmatine in an acidic microenvironment while generating large amounts of ROS through low-temperature photothermal-enhanced Fenton-like reactions, further aggravating DNA damage and significantly improving the cytotoxic effects. Both in vitro and in vivo experimental results demonstrated that P-Fe_3_O_4_@Pal@HM nanomaterials, under NIR irradiation, efficiently targeted and killed osteosarcoma cells and their HVECs. This effect was achieved by inducing apoptosis through ROS generation and RRM2 inhibition, resulting in enhanced antitumor efficacy.

Vascular damage is typically accompanied by inflammation, providing a new route for tumor treatment. This inflammatory response can be exploited to further amplify antitumor effects, making it a promising target for synergistic therapeutic strategies.^39^ We found that P-Fe_3_O_4_@Pal@HM nanomaterials, after killing osteosarcoma-associated vascular endothelial cells, can further enhance the immune response in the tumor microenvironment by promoting M1 macrophage polarization. Through Transwell coculture experiments, we confirmed that damaged endothelial cells significantly induce macrophage polarization toward the M1 phenotype, producing large amounts of proinflammatory cytokines and ROS, which further amplify the apoptotic effects on tumor cells. In in vivo experiments, we observed that P-Fe_3_O_4_@Pal@HM nanomaterials combined with NIR irradiation significantly reduced tumor volume and promoted macrophage polarization toward the M1 phenotype, further enhancing the antitumor effect. These results demonstrate that P-Fe_3_O_4_@Pal@HM nanomaterials not only directly target and kill tumor cells and vascular endothelial cells but also achieve synergistic tumor therapy by regulating the immune microenvironment. This multitargeted killing approach is a unique advantage of P-Fe_3_O_4_@Pal@HM nanomaterials, as it achieves a cascade amplification of the tumor killing effects with more lasting outcomes, theoretically offering effective prevention of tumor recurrence.

## Conclusions

In conclusion, P-Fe_3_O_4_@Pal@HM is a versatile nanomaterial that is relatively easy to synthesize and exhibits various functions. Targeting osteosarcoma tumor cells and their HVECs induces DNA damage while inhibiting RRM2 expression, preventing cellular self-repair and creating a positive feedback loop for enhanced therapy. Furthermore, the nanomaterial modulates the immune microenvironment, further amplifying the antitumor effects through a cascade mechanism. This effective, multifunctional nanomaterial offers excellent targeting and biocompatibility, presenting significant potential for clinical application in osteosarcoma treatment.

## Methods

### mRNA Sequencing Methods and the Acquisition of Genes with Differential Expression

After treatment, MNNG/HOS cells were collected and total RNA was extracted using TRIzol reagent (Invitrogen) for transcriptomic sequencing according to the manufacturer’s protocol. Pathological tissue sequencing data for osteosarcoma were obtained from the GEO (Gene Expression Omnibus) (GEO ID: GSE225588).^40^ For sequencing data with repeated samples, the “limma” package of R software was used to analyze genes with differential expression, and the Log­(fold change) (Log­(FC)) values and adjusted *P*-values were obtained for subsequent analysis after gene counting.^41^

### Single-Cell Sequencing Analysis of Osteosarcoma Tissue

We used the publicly available single-cell RNA sequencing data set GSE152048,^42^ which includes five original osteosarcoma samples (BC2, BC3, BC5, BC6, and BC16). The analysis was conducted using the Seurat package in R. Initially, the data were imported and filtered to retain cells with >200 but <2500 detected genes and with <5% mitochondrial gene expression to ensure high-quality data. The data were then normalized to account for variations in sequencing depth. High-variance genes were identified and used in subsequent steps to ensure that key biological signals were captured. Dimensionality reduction was performed using principal component analysis (PCA), followed by cell clustering using a graph-based clustering algorithm. The clusters were visualized using Uniform Manifold Approximation and Projection (UMAP) to interpret the underlying cell populations. We employed the CellChat package, which allowed us to infer and analyze intercellular signaling networks, in order to explore cell–cell communication. This involved identifying significant ligand–receptor interactions and visualizing communication patterns between different cell types, particularly focusing on the interactions between endothelial cells and macrophages to understand their role in the chemotherapy response.

### Gene Ontology (GO) Enrichment Analysis, Kyoto Encyclopedia of Genes and Genomes (KEGG) Enrichment Analysis, and Gene Set Enrichment Analysis (GSEA)

After obtaining the gene sets with differential expression, we used the GO annotations of genes in the R software package org.Hs.eg.db (Version 3.1.0) and clusterProfiler (Version 3.14.3) for the GO enrichment analysis.^43^ We used the KEGG REST API (https://www.kegg.jp/kegg/rest/keggapi.html) to obtain the latest KEGG pathway gene annotation. The R software package clusterProfiler (version 3.14.3) was used for enrichment analysis.^43^ For GSEA, we used GSEA software (version 3.0) from the GSEA Web site.^44,45^ The samples were divided into two groups based on treatment type, and the c2.cp. kegg.v7.4.symbols.gmt subset was downloaded from the Molecular Signatures Database.^46^ We combined the gene expression levels to assess the expression of related pathways. All of the analyses involved a minimum gene set of 5 and a maximum of 5000 genes. The results are presented as bubble and circle plots, as necessary.

### Correlation Expression Analysis of Two Genes and Gene Prognosis Analysis

The UCSC Xena tool was used for gene-associated expression analysis in TCGA (The Cancer Genome Atlas).^47^ GDC TCGA Sarcoma (SARC) was selected and analyzed using the HTSeq-FPKM-UQ value of the gene. We used the drawing tool that came with the Xena tool to plot figures. TCGA TARGET GTEx was obtained using UCSC Xena. We also obtained high-quality prognostic data sets for TCGA from prior TCGA prognostic studies published in Cell.^48^ Log2 (*x* + 0.001) transformation and log-rank test were used. According to relevant data, a forest plot of generalized cancer prognosis and a survival curve for single gene progression-free survival were plotted.

### Prediction of G-Quadruplex Formation According to Gene Expression Sequences

The latest annotated files of the human genome were used to obtain more accurate sequence information on the relevant genes. The promoter sequence was defined as 2000 bp upstream of the CDS. Then, the Quadruplex-forming G-Rich Sequences (QGRS) Mapper was used to predict the G-quadruplex structure.^49^ We set the maximum length of the possible G-quadruplex sequence to 45 and the minimum length to 2.

### Materials and Methods for Molecular Docking

We used AutoDock 4.2.6 software for molecular docking to confirm the binding between palmatine and G-quadruplex or double-stranded DNA.^50^ We obtained all of the DNA structures from the PDB database. Three G-quadruplex structures (PDB IDs: 1NP9,^51^ 7ClS,^52^ and 2GKU^53^) were used as receptors to demonstrate the interaction between palmatine and the G-quadruplex. Furthermore, we used four double-stranded DNA structures (PDB ID: 1G14,^54^ 1BWT,^55^ 1FZX,^54^ and 1VFC^56^) as receptors to demonstrate the interaction between palmatine and the DNA double strand. Small molecular structures such as palmatine were obtained from the ZINC database (ZINC608233),^57^ and chem3D was used for the energy minimization of MM2 to obtain more accurate results. We used AutoDock 4.2.6 or PyMOL 2.4 software^58^ to remove all of the water molecules from the ligand or receptor molecules and add all of the hydrogen atoms for molecular docking. The nonpolar hydrogen atoms were merged in AutoDock 4.2.6. The AutoDock 4.2.6 software automatically calculated the charges. The Lamarckian genetic algorithm was used for semiflexible docking. The number of GA runs was set to 100, and the optimal conformation was obtained. The AutoDock 4.2.6 software was used to plot the final docking results.

### Acquisition of Protein Interactions

We used the STRING database to analyze protein interactions and plotting with the elements provided therein.^59^ The structures and functions of the proteins were demonstrated using PDB no. 3OLJ.^60^

### Preparation of P-Fe_3_O_4_@Pal@HM Nanomaterials and Their Characteristics

We purchased palmatine hydrochloride from Shanghai Macklin Biochemical Co., Ltd.; the batch numbers, instructions, and other details regarding the drugs are available on the official Web site. We prepared P-Fe_3_O_4_ nanoparticles following the method reported by Zhu and Diao, carefully controlling the appropriate raw-material ratios and reaction time to ensure that the nanoparticle size was around 150 nm.^61^ We used high-angle annular dark-field (HAADF) imaging to confirm the correct composition of the nanoparticles. After that, palmatine was loaded onto the P-Fe_3_O_4_ nanoparticles through physical stirring. Unless otherwise specified, the loading amount of palmatine was kept the same as that in the palmatine-treated group for both the in vivo and in vitro experiments. The P-Fe_3_O_4_@Pal nanoparticles were synthesized. Successful drug loading was confirmed by infrared (IR) and ultraviolet (UV) spectroscopy. The hybrid cell membrane (HM) was prepared. The 143B human osteosarcoma cells were cultured stably in vitro, and osteosarcoma-associated heterogeneous vascular endothelial cells (HVECs) were obtained using a coculture method through a Transwell system. The cell membranes from both cell types were extracted and mixed at a 1:1 mass ratio to form the HM. The HM was then mixed with the P-Fe_3_O_4_@Pal nanoparticles, and the mixture was passed through an extruder to generate P-Fe_3_O_4_@Pal@HM nanomaterials. The pore size of the polycarbonate membrane in the extruder was 200 nm. The extrusion process involved at least 60 repetitions at low temperatures to ensure that the membrane structure remained intact. We used genetic engineering techniques to introduce Flag and His tags onto the surfaces of the two cell membrane types in order to verify the membrane composition via Western blotting. These tags were then detected by Western blotting (detailed steps are shown in the schematic in the Results section). In addition, we confirmed the accuracy of membrane hybridization using fluorescent-dot-blot hybridization assays. Finally, we validated the structural integrity of the nanomaterials using transmission electron microscopy (TEM) and measured the nanoparticle size and zeta potential using a Malvern Zetasizer Nano ZS90. When cells were treated with the nanomaterials, their concentration was calculated based on the content of P-Fe_3_O_4_. Nanomaterials containing 100 μg/mL of P-Fe_3_O_4_ were used in in vitro cell experiments and characterization analyses unless otherwise specified.

### Drug Loading and Drug Release Efficiency

Palmatine was adsorbed onto P-Fe_3_O_4_ during the synthesis of the nanomaterials. An excess amount of the drug (four times the mass of the nanomaterial) was mixed with P-Fe_3_O_4_ at ambient temperature overnight. The next day, the nanomaterial was separated by centrifugation, and the supernatant was collected to determine the drug content. The quantity of drug loaded onto the nanomaterial was determined by subtracting the amount of drug found in the supernatant from the total amount of drug used. The formula for calculating the drug loading capacity was as follows:

$$\mathrm{Drug}\;\mathrm{loading}\;\mathrm{efficiency}\left(\%\right)={\frac{\mathrm{Weight}\;\mathrm{of}\;\mathrm{loaded}\;\mathrm{drug}\left(\mu g\right)}{\mathrm{Weight}\;\mathrm{of}\;\mathrm{nanomaterial}\left(\mu g\right)}}^{*}100\%$$

The drug release rate was assessed at pH 7.4, pH 5.0, pH 7.4 + 100 μM H_2_O_2_ + NIR, and pH 5.0 + 100 μM H_2_O_2_ + NIR. We placed P-Fe_3_O_4_@Palmatine@HM (containing 100 μg/mL of P-Fe_3_O_4_) in different environments. A small volume of the solution was removed after 3, 6, 12, 24, 36, and 48 h. After centrifugation, the supernatant was collected to measure the drug release levels. The drug release rate was calculated using the following formula:

$$\mathrm{Drug}\;\mathrm{releasing}\;\mathrm{efficiency}\left(\%\right)={\frac{\mathrm{Weight}\;\mathrm{of}\;\mathrm{released}\;\mathrm{drug}\left(\mu g\right)}{\mathrm{Weight}\;\mathrm{of}\;\mathrm{loaded}\;\mathrm{drug}\left(\mu g\right)}}^{*}100\%$$

### Cell Line Culture and Treatment

The 143B osteosarcoma cell line (Cat# CL-1031) was obtained from Procell Life Science & Technology Co., Ltd. and cultured in Minimum Essential Medium (MEM) supplemented with 10% fetal bovine serum (FBS) and 100 U/mL penicillin-streptomycin (final culture medium, also from Procell). Human umbilical vein endothelial cells (HuVECs) and THP-1 human monocytic cells were cultured in media recommended by their respective protocols. THP-1 cells were differentiated into M0 macrophages by treatment with 0.1 μg/mL phorbol 12-myristate 13-acetate (PMA). Unless otherwise stated, all cells were cultured in a humidified incubator at 37°C with 5% CO_2_. Serum-free cell cryopreservation medium (CELLSAVING, Cat# C40100/C40050) was purchased from New Cell & Molecular Biotech Co., Ltd. Disposable nitrile gloves and T25 cell culture flasks were supplied by ABclonal Biotechnology Co., Ltd.

### Detection of the Targeting and Cytophagocytosis Ability of P-Fe_3_O_4_@Pal@HM Nanomaterials

We observed the membrane fusion and phagocytosis of nanomaterials using confocal microscopy (Olympus, Japan) and confirmed the interactions between nanomaterials and osteosarcoma cells/HVECs using flow cytometry. To evaluate the affinity between osteosarcoma cells/HVECs and nanomaterials, we labeled the cell membranes of osteoblasts, osteoclasts, bone mesenchymal stem cells (BMSCs), osteosarcoma cells, HVECs, and chondrocytes with various combinations of DiI, DiD, and Hoechst 33342. The specific grouping method is detailed in the flowchart within the Results section. After labeling, the cells were digested, resuspended, mixed, and cocultured. Once the cells were stably attached, nanomaterials at a concentration of 100 μg/mL of P-Fe_3_O_4_@Pal@HM were introduced and cocultured for 6 h. Because palmatine binds to DNA and emits green fluorescence (aggregation-induced emission^62^), it was detected using the FITC channel. Following coculture, the medium was discarded, the cells were washed with phosphate-buffered saline (PBS), and flow cytometry was performed. Confocal microscopy was used to observe the phagocytosis and membrane fusion of the nanomaterials. Due to the need for DiO or FITC Phalloidin for membrane or cytoskeleton labeling and to avoid fluorescence interference from palmatine, we designed DiI-labeled nanomaterials, P-Fe_3_O_4_@HM-DiI, to observe the membrane fusion processes. In addition, we designed P-Fe_3_O_4_@ICG@HM nanomaterials to observe the phagocytosis of the nanomaterials by cells. Because the targeting ability of nanomaterials depends on HM, P-Fe_3_O_4_@ICG@HM nanomaterials can truly reflect the targeting ability of P-Fe_3_O_4_@Pal@HM. We cocultured P-Fe_3_O_4_@HM-DiI nanomaterials with osteosarcoma cells or HVECs, as outlined in the Results section. Following coculture, the medium was removed, and cells were washed with PBS, fixed with 4% paraformaldehyde, and permeabilized using PBS containing 0.5% Triton X-100. FITC Phalloidin (purchased from Beijing Solarbio Science & Technology Co., Ltd, Cat# CA1620) and DAPI staining was performed followed by confocal microscopy. In addition, we confirmed the interaction between nanomaterials and osteosarcoma cells or HVECs through membrane fusion. As mentioned above, DiI was used to label HMs and DiO to label the cell membranes of osteosarcoma cells or HVECs. Confocal microscopy was used to detect red fluorescence on the cell membrane (green fluorescence) after coculture. Furthermore, we validated the in vivo targeting of the nanoparticles by injecting mice with P-Fe_3_O_4_@ICG@HM. Organ imaging in vivo was conducted using Bruker’s in vivo FX PRO at the time of nanomaterial injection and at 3 and 6 h postinjection.

### Biocompatibility Test of the P-Fe_3_O_4_@Pal@HM Nanomaterials

We conducted a hemolysis test to assess the circulatory toxicity of the nanomaterial. The P-Fe_3_O_4_@Pal@HM nanomaterial was prepared at various concentrations, with the maximum concentration containing 200 μg/mL P-Fe_3_O_4_. Red blood cells (RBCs) were isolated from fresh blood and washed with PBS. The RBCs were then incubated with different concentrations of the nanomaterial for 4 h at 37 °C. Following incubation, the samples were centrifuged, and the supernatant was collected to measure absorbance at 541 nm using a microplate reader. We next evaluated the toxicity of the P-Fe_3_O_4_@Pal@HM nanomaterial in normal bone tissue cells to ensure it did not compromise their viability while targeting osteosarcoma cells. Osteoblasts, chondrocytes, osteoclasts, and bone marrow mesenchymal stem cells (BMSCs) were cultured in appropriate media. The cells were then exposed to various concentrations of the nanomaterial for 24 h. Cell viability was assessed using the Cell Counting Kit-8 (CCK-8) assay. In brief, CCK-8 solution was added to every well, and the plates were incubated for an additional 2 h. The absorbance was measured at 450 nm using a microplate reader. In vivo experiments were conducted on healthy mice, with nanomaterials administered via the caudal vein, in order to examine the systemic organ toxicity of the nanomaterial. After the respective treatments, the mice were euthanized and the major organs, namely, the heart, liver, spleen, lung, and kidney, were harvested. The organs were fixed in formalin, embedded in paraffin, sectioned, and stained with hematoxylin and eosin (HE) for histological examination. The tissue morphology was evaluated under a microscope to assess any pathological changes.

### Photothermal Performance of P-Fe_3_O_4_@Pal@HM Nanomaterials

The photothermal effects of nanomaterials were assessed in vitro and in vivo. A NIR laser with a wavelength of 808 nm and a power density of 1.8 W/cm^2^ was used. Temperature measurements were performed using an infrared temperature detector. We initially prepared PBS, P-Fe_3_O_4_, P-Fe_3_O_4_@Pal, and P-Fe_3_O_4_@Pal@HM (containing 100 μg/mL P-Fe_3_O_4_) and exposed them to NIR irradiation for 300 s. Temperature readings were taken every 30 s. After that, different concentrations of P-Fe_3_O_4_@Pal@HM (containing 25, 50, 100, and 200 μg/mL P-Fe_3_O_4_) in PBS were prepared and irradiated with the NIR laser for 300 s, with temperature measurements taken at 30 s intervals. P- Fe_3_O_4_@palmatine@HM (containing 100 μg/mL of P-Fe_3_O_4_) was irradiated for 300 s, with temperature recordings every 30 s, to verify the photothermal stability of the nanomaterials. After stopping the NIR irradiation, the nanomaterial was allowed to cool naturally, and the temperature was recorded every 30 s for 570 s. After cooling to room temperature, the nanomaterial was reirradiated. Xenografts in nude mice were irradiated after injection of nanomaterials containing 10 mg/kg P-Fe_3_O_4_ via the tail vein, for in vivo photothermal stability. Eight hours after injection, the xenografts were irradiated for 300 s and then allowed to cool naturally, with the average temperature recorded every 30 s and a temperature–time curve plotted. Throughout the study, cells or tumor tissues were maintained at 42.5 ± 0.5 °C via NIR irradiation, both in vitro and in vivo, for a duration of 5 min, unless otherwise specified.

### Flow Cytometry of Apoptosis

An annexin V-PE/7AAD apoptosis kit was selected because we considered the fluorescence presented by the drug molecule binding to DNA. We followed the specific steps outlined in the manufacturer’s instructions. The flow cytometer was a BD Accuri C6 Plus, while FlowJo VX software was used to analyze the flow cytometry results of apoptosis.

### Flow Cytometry Analysis of Macrophage Polarization

In this study, macrophages were prepared for flow cytometry to analyze their polarization. Initially, the Fc receptors on the macrophage surfaces were blocked by incubating the cells with an anti-CD32 antibody. After blocking, the cells were washed thoroughly and then incubated for 30 min at room temperature with fluorescently labeled anti-CD11b and anti-CD86 antibodies. All of the staining procedures were performed in the dark. Following staining, the cells were washed again and the expression levels of CD11b and CD86 were measured using flow cytometry.

### CCK-8 Assay

The cells in the culture flask were digested, suspended, and evenly placed in a 96-well plate, with every well containing 5000 cells. After cell adherence, the complete culture medium containing different concentrations of the drugs was replaced. After coculture, the cell viability was detected using a CCK-8 assay kit (Dojindo Laboratories, Japan). Every group included six replicates.

### Western Blot Analysis

Sodium dodecyl sulfate-polyacrylamide gel electrophoresis (SDS-PAGE) was used to detect the expression of the corresponding proteins in cells. The appropriate gel concentration was selected based on the protein molecular weight. First, radioimmunoprecipitation assay (RIPA) lysis buffer was used to lyse the cells and extract proteins. The protein concentration was determined using a bicinchoninic acid assay (BCA) protein concentration determination kit (Vazyme Biotech Co., Ltd.). Then, protease inhibitors and loading buffer with an appropriate volume were added, and the mixture was evenly mixed and heated at 95 °C for 20 min to denature the protein. After that, sample loading and electrophoresis were performed. The polyvinylidene fluoride (PVDF) membrane was used for membrane transfer. We set the membrane transfer current to 200 mA and the membrane transfer time to 10 m. After membrane transfer, 5% skim milk solution was used to block for 1 h at room temperature. The PVDF membrane was then coincubated with the primary antibody at 4 °C overnight. After incubation, the bands were washed with triethanolamine-buffered saline solution containing Tween 20 (TBST) and then coincubated with secondary antibodies at room temperature for 1 h. After washing with TBST, the membrane was developed using an enhanced chemiluminescence (ECL) solution (Vazyme Biotech Co., Ltd.).

### Animal Experiments

All of the animal experiments were performed in compliance with the guidelines of the Ethics Committee of the Experimental Animal Center at Huazhong University of Science and Technology, China. We used a xenograft tumor model in BALB/c nude mice. Every animal received an injection of 1.5 × 10^7^ 143B osteosarcoma cells in 150 μL of solution near the groin of the right lower limb. In the control group, tumors were injected with PBS only. Nanomaterials were administered via tail vein injection, with dosages typically calculated based on the P-Fe_3_O_4_ content (10 mg/kg) unless otherwise specified. The detailed groupings are illustrated in the Results section. The methods for assessing in vivo photothermal performance, safety, and targeting were previously described. A total of four injections were administered, with every injection being spaced 3 days apart. On the second day following the final injection, the tumors were excised and measured. Every treatment group included three replicates. *K*_i_-67 and immunofluorescence staining were performed to analyze cell proliferation and the expression of key proteins within xenograft tumors.

### Statistical Analysis

We used analysis of variance (ANOVA) to analyze the statistical differences between groups. A *P*-value <0.05 indicated a statistical significance. We used GraphPad Prism 8 to perform statistical tests and plot statistical figures.

## Supplementary Material



## Notes

Single-cell sequencing results, GO enrichment analysis, statistical analyses of Western blot results, correlation studies based on TCGA data, cell cycle impact assessments, effects of cisplatin on RRM2 expression, promoter sequence predictions, gene expression heatmaps, fluorescence accumulation data, nanomaterial phagocytosis and TEM observations, and ELISA results from macrophage coculture experiments (PDF)
