# Supplementary material for: Immune-Modulating Dual-Targeted Nanomaterials for Low-Temperature Photothermal–Photodynamic–Chemodynamic Therapy of Osteosarcoma Targeting Tumor and Endothelial Cells
Source: ACS Nano. 2025 Aug 20;19(34):30786–809. doi: 10.1021/acsnano.5c00223 (PMC12410057; doi:10.1021/acsnano.5c00223)

Supplementary Information

Immune-Modulating Dual-Targeted Nanomaterials for Low-Temperature Photothermal-Photodynamic-Chemodynamic Therapy of Osteosarcoma Targeting Tumor and Endothelial Cells

Qing Pan[1, #], Wei Wu [1, #], Doudou Jing[2, #], Wei Huang[1], Yongzhi Cui[3], Zhicai Zhang[1], Zengwu Shao[1, *], Hongzhi Hu[1, *], Wenbo Yang[1, *]

1. Department of Orthopaedics, Union Hospital, Tongji Medical College, Huazhong University of Science and Technology, Wuhan, 430022, China
2. Department of Orthopaedics, Shanxi Medical University Second Affiliated Hospital, Taiyuan, Shanxi, 030001, China
3. Department of Orthopaedics, Shanghai Sixth People's Hospital Affiliated to Shanghai Jiao Tong University School of Medicine, Shanghai, 200080, China.

#: These authors contributed equally.

*: To whom correspondence should be addressed:

mail: yangwenbohust@163.com, 13720105746@163.com and szwpro@163.com

Figure S1. Single-cell sequencing results of osteosarcoma tissue, displaying different cell types.

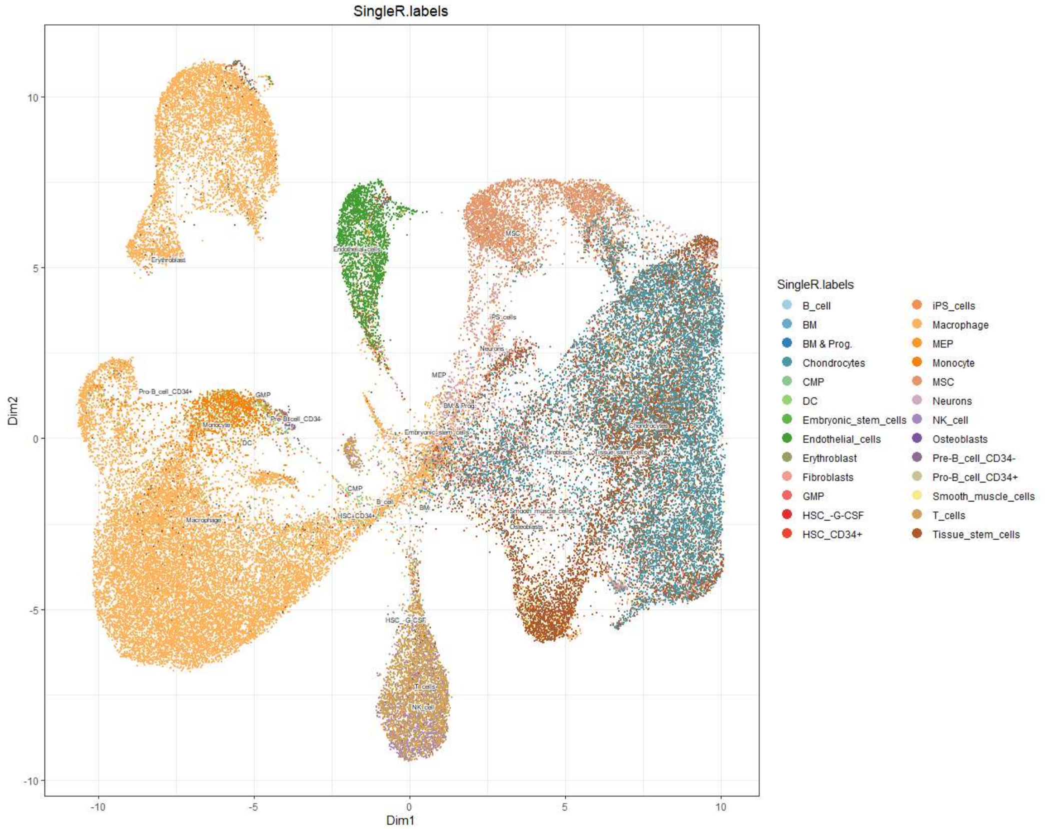

Figure S2. Circle plot depicting predicted intercellular regulatory crosstalk between endothelial cells (ECs) and diverse immune cell subsets within osteosarcoma tissue. Chord widths are proportional to the number (and strength) of predicted ligand–receptor interactions.

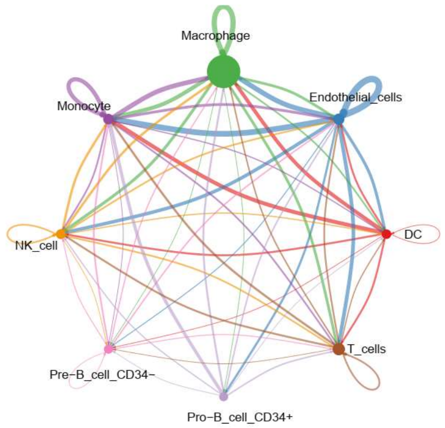

Figure S3. GO (Gene Ontology) biological process enrichment analysis results of differentially expressed markers between osteosarcoma-associated heterogeneous vascular endothelial cells and normal vascular endothelial cells.

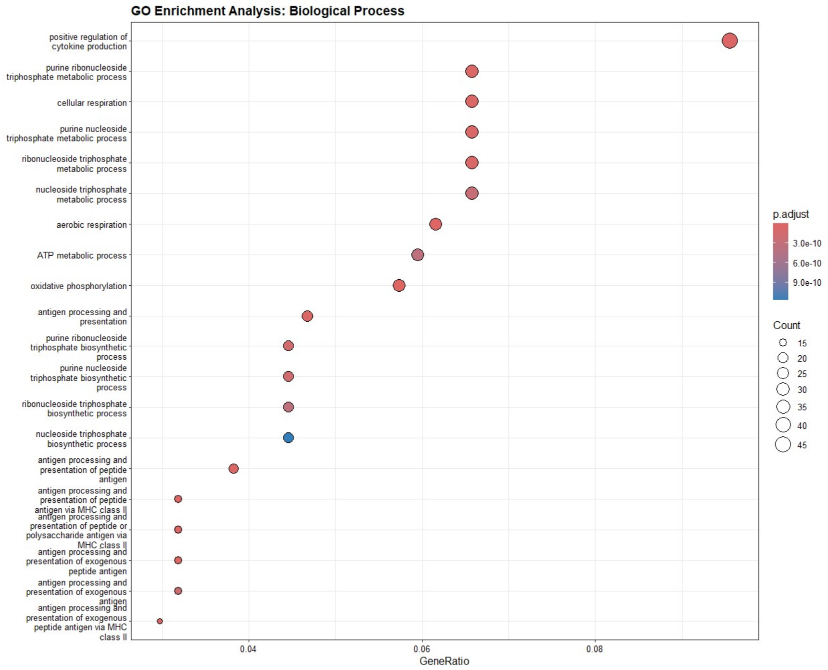

Figure S4. Statistical analysis results of western blot for the expression of cell cycle and DNA damage repair-related proteins cyclinB1 and CHEK1 in osteosarcoma cells and HVECs after RRM2 overexpression. ns: Non-significant. $^{*}p < 0.05$; $^{**}p < 0.01$; $^{***}p < 0.001$.

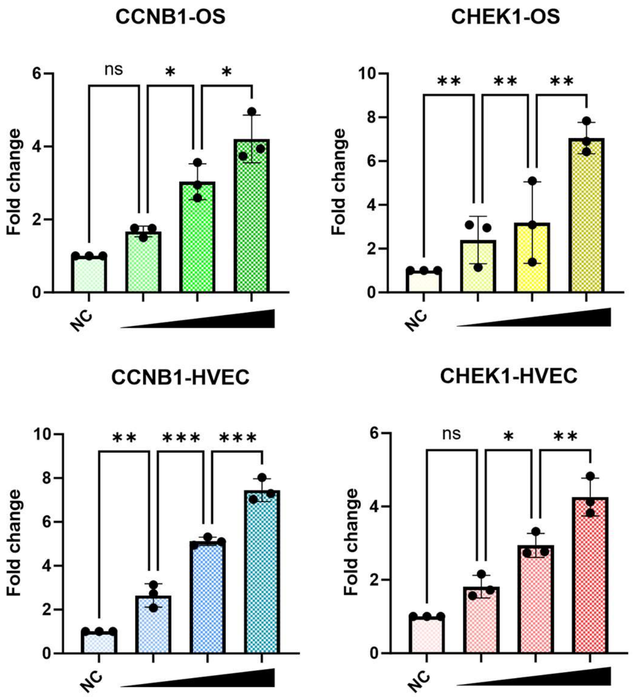

Figure S5. Correlation between RRM2 expression and cyclinB1, cyclinE1, CHEK1, and TP53 expression: data based on TCGA sarcoma samples.

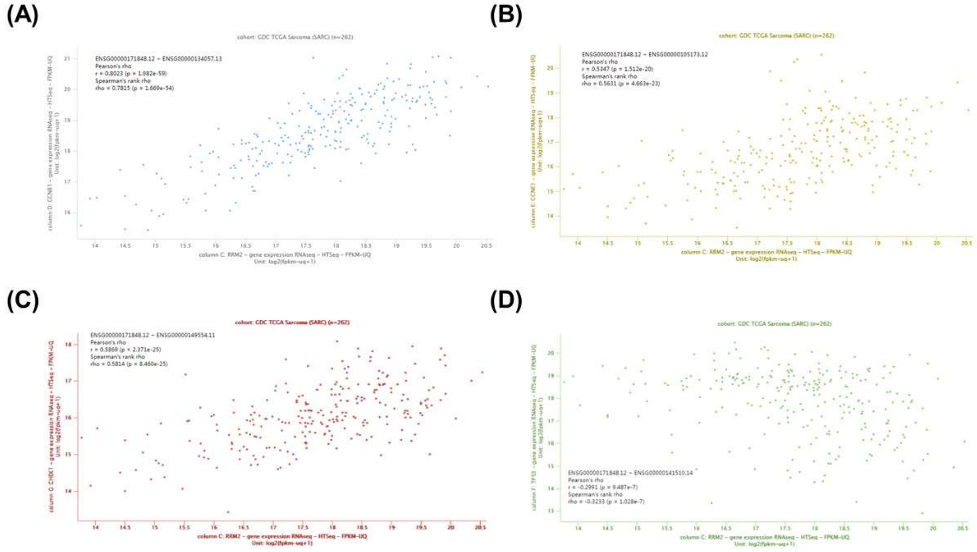

Figure S6. Association between RRM2 expression and clinical outcomes: (A) new tumor event after initial treatment and (B) metastatic diagnosis. RRM2 expression is significantly higher in patients with new tumor events and metastasis ($p < 0.05$), suggesting its potential role in tumor progression.

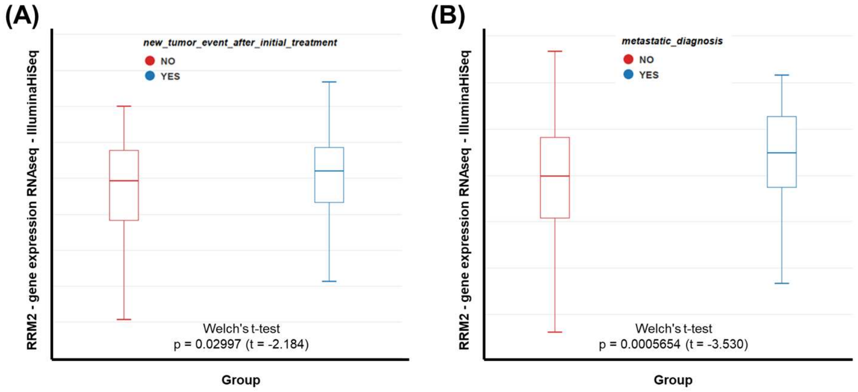

Figure S7. Effect of different treatments on the cell cycle of osteosarcoma-associated heterogeneous vascular endothelial cells and statistical analysis results. *p < 0.05; **p < 0.001.

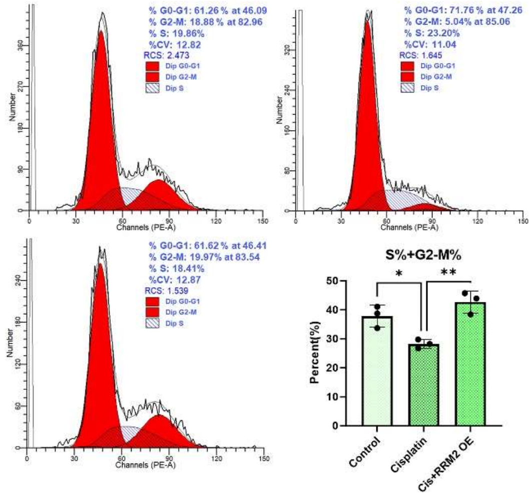

Figure S8. Western blot results and statistical analysis of the effects of different concentrations of cisplatin on RRM2 expression levels in osteosarcoma cells and osteosarcoma-associated heterogeneous vascular endothelial cells. ****p < 0.0001.

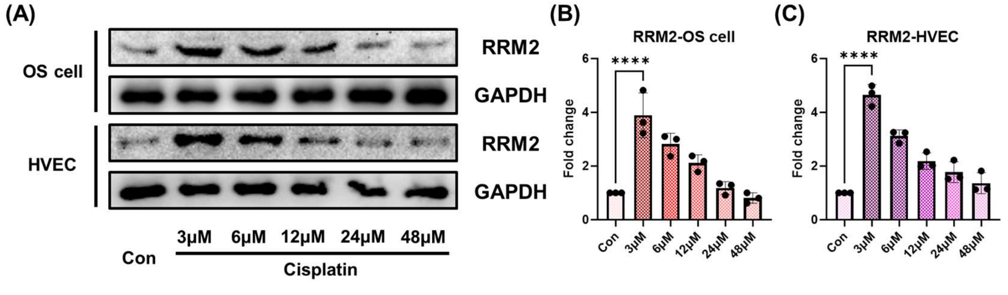

Figure S9. Prediction results of the RRM2 promoter sequence based on the NCBI database. Highlighted regions indicate sequences likely to form G-quadruplex structures.

```
000001 GTATGAAGTC TTCTTGCCAA TAACTTTTAG CCTTAGGCTA ATAACGATCC CCCGCCGGAC TTTTTTTTTT TTTTTTGAGA CGGAATTTCA TTCTTGTTGC
000101 CCAGGTTGGA GTGCAATGGT GTGATCTCGG CTCACTGAAA CCTCTGCCTC CTGGGTTCAA ATGATTCTCC TGCCTCAGCC TCCCAAGTAG CGGGAACCCA
000201 AGTAGCGGGA ATTACAGGCG CCTGCTGCCA CGTCTGGCTA ATTTTTGTAT TTTTAGTAGA GATGGGGTTT CCCTATATTG ACCAGGCTGC TCTCAAACTC
000301 CTGACCTCAG GTGATCTGCC TGCCTCACCC TCCCAAAGTG CTGGGATTAC AGGCGTGAAC CTCCAAGCAG GGCCTAATAA AGCCTTTTGA CCTAACTTCC
000401 AGTGTAGAGT ACAGGGGTCA GGAAAACAAG TTCATTGACA CTGTGAATAA ACAATTAGGC AAATGTAGAA TGTGGGACAT TCTACAAGAA AACTGGCCTG
000501 TACTTCAAAG TCAATGTCGT GGGGAAAAGA AGTGGAGGAG CTTTACTAAG AGTCTAAAGC AACATAATAA ACGCAATGCA TGTGATTAAT TAGATACTGC
000601 ACAAAAAAAT AAATCCAAAA AACTATGAGA CATTTTTGAA ACAATTGGAA AAACAGGAAT ATGGACTGGA TATTAGGTTA TATTAGAGAA TTATTGTTAG
000701 TTTTCCTAGG GATAAATGTG GTATTGTGGT GATATTAACA GAGTGTCCTA ATTTTTAGGA GCTGAAACGT TTAGGGGTGA TGGGTTATGA TATCTATCAC
000801 TCACTTTCAA ATGATCCATA AAAAAGCATA AGCCTCATAC ACCCACAGAG GGGGTGTCTG CCACATTTGC CATGAAGCAA ATATGGCAAA ATGGAAACAG
000901 CTGTTGAATC CTAGCTGGTG GGTGAACACT AGGAATCCTT CCATTATTCT GTATGTTGAA AACATTCATA GTAAAAAGCT AGGCTAAAAA CGTTCTGCTG
001001 TGCAGTTGTG CTGTGCGACC TTGGGCAGTT TCCTTGCCTC CCAGGCCTTC ATTTCATCAC CTATGAAAAG GGTTAAAAAT ACCAACCCCG GTGGGGCGGT
001101 GGCTCACGCC TGTAATCTGA GCATTTTGGG AGGCCAAGGC GGGTGGATGG CTTGAGCCCA GGGGTTCAAG ACCAGCCCGG GTAACATACC GAGAACCCAT
001201 CTCTACAAAA AATTAAAAAA TTAGCCTGGC GTAGTGGTGC CTGCCTTGAG TCCCAGCTAC TCGGGAGGCT GAGGTGGGAG GATCGCTTGA GCTCGGATAG
001301 TTGAGGCTGC AGTGAGCTAT CATGGAGCCA CCAGACTGGG CAACAGAGCA AGACCCTGTC TCAAAACAAA AAACAAAAAA CTCAAACAGC CTCACATGAT
001401 CCAGGCGCCC AGAGGCTGTG GGTGCTGCAG CCTTAATTAT TATTATGACC ACCTTCGGCA CGGCGTCTGG TACACAGTGA GTGCTGAGCT CCCCGAGCCG
001501 CGGTTTCTCC ACCCTAATGG TGAACAGCCT TTTGGAAGTC GCGCTAACCT TGGCCTGAGA CCTGCAAACT TGCCCAGGCT GGGGCGTGTG AACCGGCGAG
001601 CGCGCAGCGG AAACGGGGCG GGGCACCTGA GGCTGGGAAT GCAGAGGAGC CTTCCGGGGG GCGGGGCGGG GCCTCCCGTG CAGACCAATG GTGGAGTAGA
001701 TGCAGATGTC AAAACGCGCG CTCAAGTGGC TTCCGCCAGG AATCCCGACG CTTAGGGAGG CGGAGGGAGG ATCGCTTGAG ACCAGCCTGG GCAAACAAGC
001801 GAGACCCTCG TCTGTTTACT TAAATAAAAC CAAAAAAACG AGCACCGAGG GAAAAAGGAG TGAATCCCGG GGCTAGCAGC AGCCTGCGGC GGGCGCTCTC
001901 CCGGGAGTGG CTGCACCGCC CGACCTCCCC GGAGGCGGAA CCGCCCGCAT TGCCGCGTGG CCCTGGGCGC CGCCACCTCC TCCGCAGCGG GGCAAAGTTG
```

Figure S10. Heatmap of key differentially expressed genes in osteosarcoma cells after palmatine treatment.

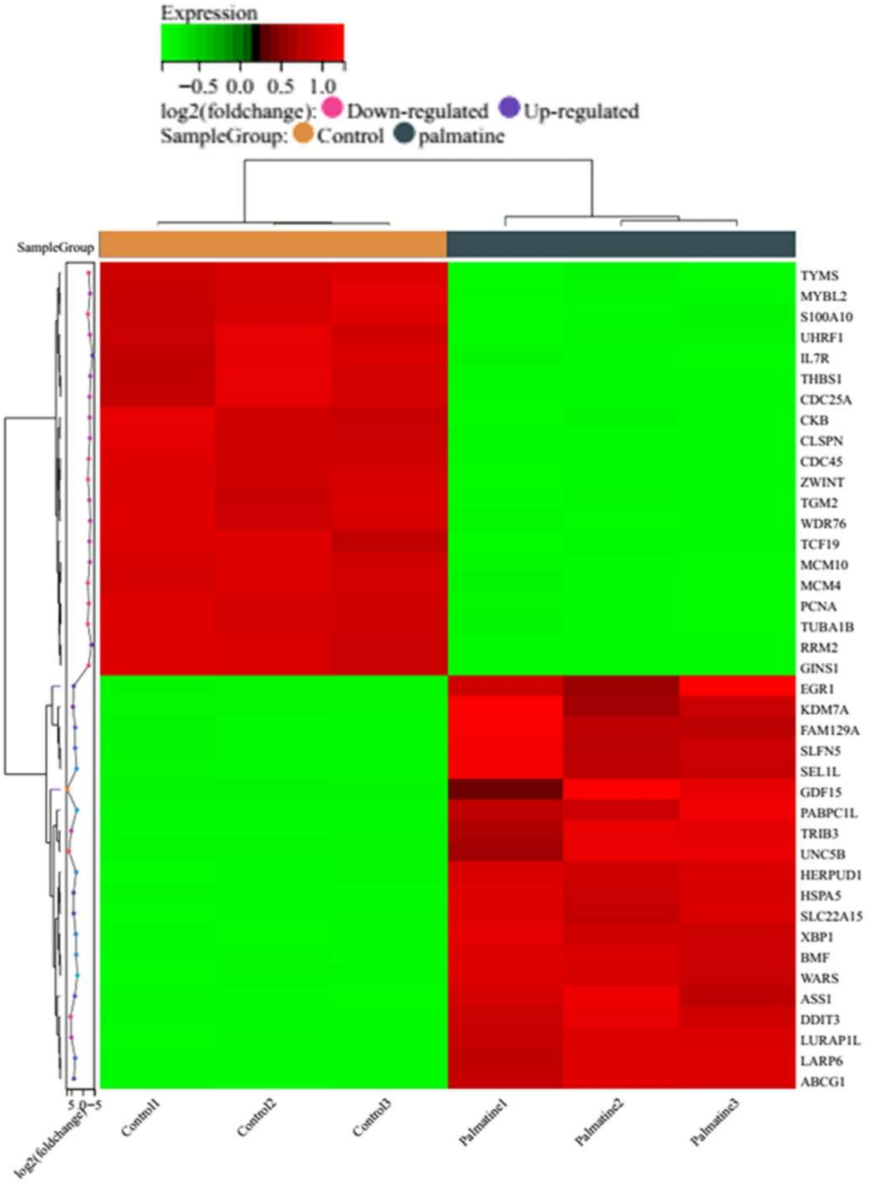

Figure S11. Statistical analysis of fluorescence accumulation from flow cytometry results. Ns: Non-

significant. ****$p < 0.0001$.

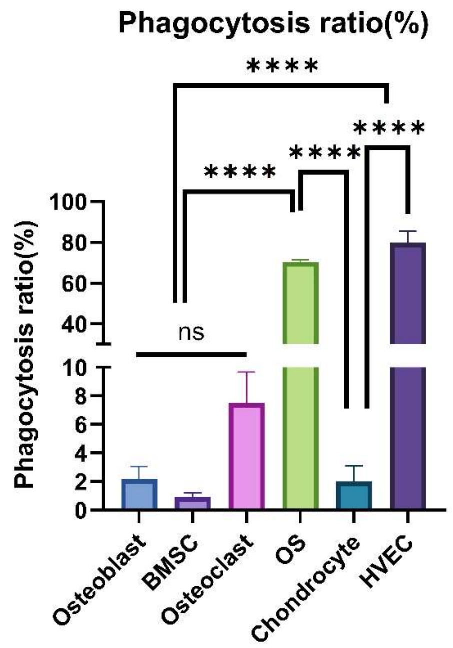

Figure S12. Phagocytosis of P-Fe_3O_4@ICG@HM by osteosarcoma cells and osteosarcoma-associated heterogeneous vascular endothelial cells, further confirming the internalization of nanomaterials.

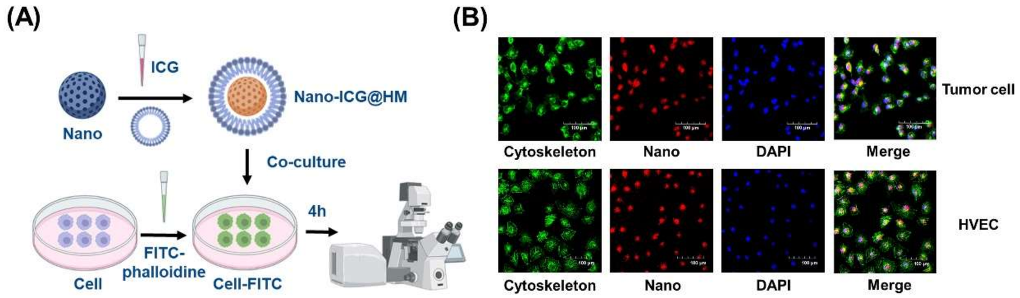

Figure S13. Internalization of P-Fe_3O_4@ICG nanoparticles. Without HM coating, the uptake of the nanoparticles by tumor cells or HVECs is quite limited.

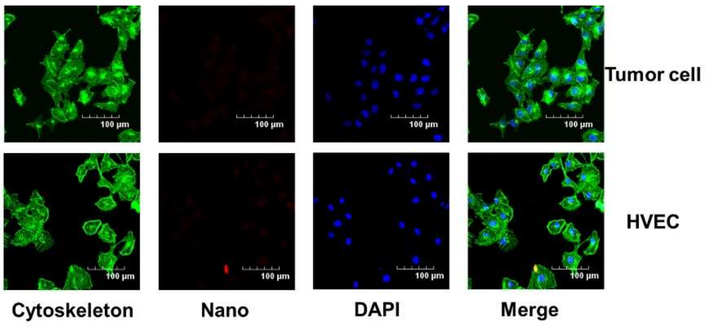

Figure S14. TEM results of nanomaterials after low-temperature photothermal treatment.

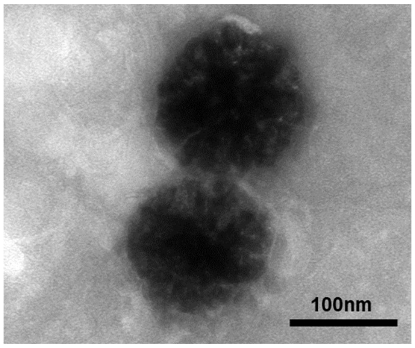

Figure S15. TMB assay verifying the Fenton-like reaction under different conditions. (A) Under acidic conditions (pH=5.0), the catalytic activity varies with nanomaterial composition and is significantly enhanced by NIR irradiation, indicating the importance of both nanoparticle components and photothermal activation. (B) Stronger catalytic activity is observed with decreasing pH, demonstrating the crucial role of the acidic tumor microenvironment in promoting the Fenton-like reaction. ***p < 0.001; ****p < 0.0001.

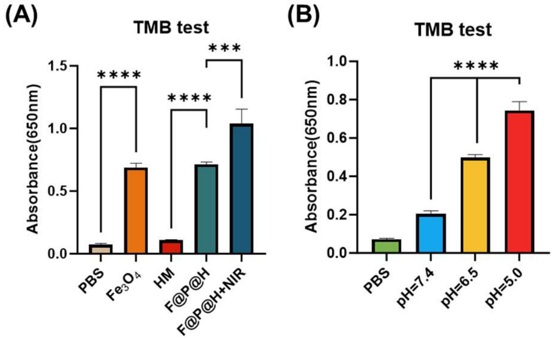

Figure S16. Effect of P-Fe_3O_4@Pal@HM on homologous recombination (HR) DNA repair. The results indicate that palmatine (Pal) is a key component in inhibiting DNA damage repair, and its suppression of homologous recombination may further enhance the targeted cytotoxicity of the nanoparticles. **p < 0.01; ***p < 0.001; ****p < 0.0001.

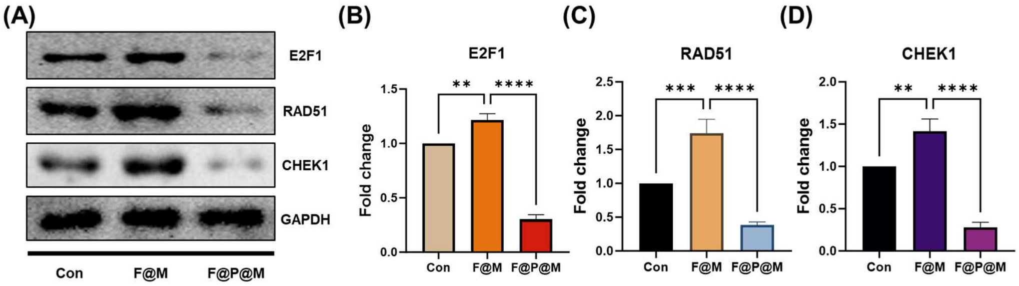

Figure S17. Western blot results and statistical analysis of TP53 and RRM2 expression levels in osteosarcoma cells and osteosarcoma-associated vascular endothelial cells after different treatments. A. Control, B. P-Fe_3O_4@Pal@HM+NIR, C. NAC; D, P-Fe_3O_4@Pal@HM+NIR+NAC. *p < 0.05; **p < 0.01; ***p < 0.001; ****p < 0.0001.

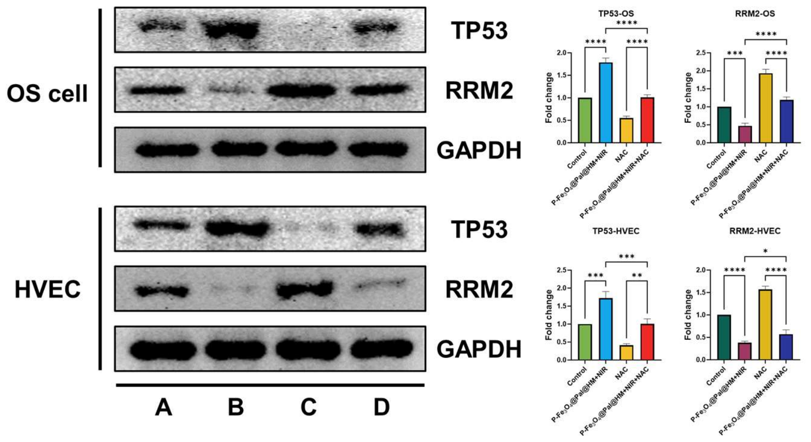

Figure S18. ELISA results of antitumor factor expression levels in macrophages after co-culture with different cell types. ns: Non-significant. ****$p < 0.0001$.

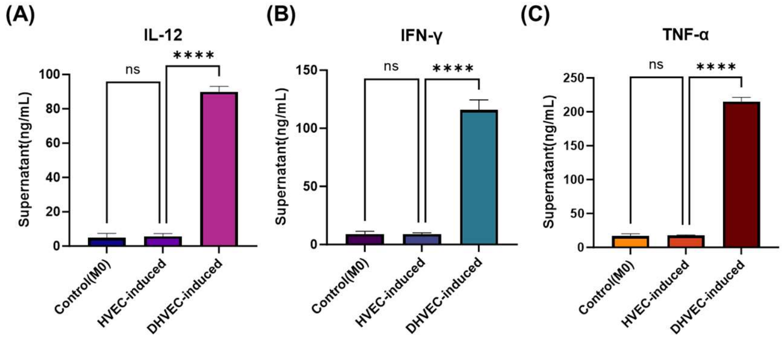

Supplement: Supplementary file 1 [file nn5c00223_si_001.pdf]
